# Prevalence and gender‐specific correlates of hazardous and binge drinking among Swedish and Finnish older adults

**DOI:** 10.1111/acer.70098

**Published:** 2025-08-01

**Authors:** Wossenseged Birhane Jemberie, Johan Niklasson, Knut Lönnroth, Erika Boman

**Affiliations:** ^1^ Department of Social Work Umeå University Umeå Sweden; ^2^ Centre for Demography and Aging Research (CEDAR) Umeå University Umeå Sweden; ^3^ Department of Community Medicine and Rehabilitation, Geriatric Medicine, Sunderby Research Unit Umeå University Umeå Sweden; ^4^ Department of Global Public Health Karolinska Institutet Stockholm Sweden; ^5^ Government of Åland Mariehamn, Åland Finland; ^6^ Department of Nursing Umeå University Umeå Sweden; ^7^ Department of Research and Development Åland University of Applied Sciences Mariehamn, Åland Finland

**Keywords:** alcohol use, epidemiology, gender, older adults, risk factors

## Abstract

**Background:**

Alcohol consumption is a leading modifiable risk factor for a range of diseases and social harms globally. Older adults are vulnerable to alcohol‐related harms due to physiological changes, multimorbidity, and medication use; however, many older adults continue to drink at high‐risk levels. This study examined the prevalence and gender‐specific correlates of hazardous and heavy episodic drinking (HED) among Swedish and Finnish community‐dwelling older adults.

**Methods:**

Cross‐sectional data from the 2021/2022 Gerontological Regional Database (GERDA) survey included 11,747 participants aged 65, 70, 75, 80, 85 and 90 years. Missing data were multiple imputed by chained equations. Hazardous drinking was defined as an AUDIT‐C score of four or more, and HED was defined as consuming six or more drinks on a single occasion at least monthly. Sociodemographic, psychosocial, functional status, and health‐related factors were analyzed using multinomial and logistic regression models, stratified by gender and accounting for regional differences.

**Results:**

Overall, 30.2% (95% CI, 29.0–31.4) of men and 9.8% (95% CI, 9.1–10.6) of women were classified as hazardous drinkers. HED prevalence was 13.0% (95% CI, 12.1–13.9) in men and 2.9% (95% CI, 2.5–3.3) in women. Hazardous drinking and HED in women were associated with higher socioeconomic status and psychosocial stressors, such as depression and bereavement, while functional and health‐related factors were significant predictors of problematic alcohol use in men. Across both genders, religious participation was a protective factor, while self‐reported cardiovascular disease was associated with increased risk of hazardous drinking.

**Conclusions:**

Hazardous and are prevalent among older adults in Sweden and Finland with some regional differences, and notable gender differences in associated risk factors. There is a need for interventions that focus on strengthening resilience to psychosocial stressors and provide older adults with clear, consistent health communication about alcohol's harmful effects on cardiovascular and overall health.

## Graphical abstract

This study found a high prevalence of hazardous and heavy episodic drinking among older adults in Sweden and Finland, with notable gender and regional variations. Multivariate analyses showed men's drinking patterns correlated with functional and health‐related factors, while women's drinking patterns were associated with psychosocial factors, including depressive symptoms and bereavement. Religious participation was a protective factor across both sexes. There is a need for interventions that strengthen psychosocial resilience and provide clear communication on alcohol's harmful effects on cardiovascular and overall health.

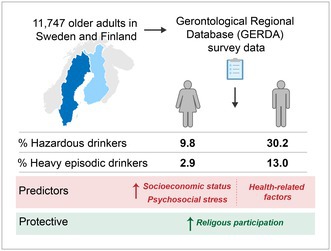

## INTRODUCTION

Alcohol consumption is one of the leading modifiable risk factors for a wide range of noncommunicable diseases, mental health conditions, injuries, and social harms globally (Klingemann & Gmel, 2001; Rehm et al., 2017). With advancing age, even alcohol consumption levels considered low‐risk for younger populations can pose significant health burdens. Changes in metabolism, body composition, and organ function, combined with multimorbidity and increased medication use, heighten individuals' susceptibility to alcohol‐related harms in later life (White et al., 2023). Older adults are particularly vulnerable to acute risks, such as falls, injuries, and severe drug‐alcohol interactions, as well as chronic conditions such as cardiovascular disease, liver cirrhosis, and cognitive decline (White et al., 2023). Nevertheless, many older adults continue to drink alcohol, often at levels that exceed low‐risk drinking recommendations, due to perceived social, psychological, and health‐related benefits (Immonen et al., 2011a; Jemberie et al., 2023).

While alcohol use typically declines with age, recent cohorts of older adults, especially women, present higher and more frequent alcohol consumption compared to previous generations (Ahlner et al., 2018; Bryazka et al., 2022; Grucza et al., 2018; Moore et al., 2005). More than one‐third of older adults in Nordic and other Western countries now engage in at‐risk drinking (Kuerbis, 2020; Ramstedt & Guttormsson, 2024; Stelander et al., 2022). Moreover, as the number of very old adults (80+ years old) continues to rise, even low prevalence rates of alcohol use may result in a growing public health burden, highlighting the importance of monitoring drinking patterns in this age group. However, relatively few studies have included very old adults, despite this demographic segment being one of the fastest‐growing across high‐income countries, leaving a gap in knowledge about late‐life alcohol use patterns and associated factors.

Prior research has identified various demographic, psychosocial, and health‐related factors associated with problematic alcohol use (Kuerbis, 2020), yet findings remain inconsistent due to limited cross‐national data with comparable cohorts and insufficient gender‐stratified analyses. Men and women differ in their drinking patterns, risk factors, and the social and health consequences they experience (Bryazka et al., 2022; Maxwell et al., 2022). Understanding these differences is essential for designing targeted interventions, such as screening and risk communication. This study examines the prevalence and gender‐specific correlates of hazardous and heavy episodic drinking (HED) among Swedish and Finnish community‐dwelling older and very old adults.

### Study context

Until recent years, Sweden and Finland maintained generally restrictive alcohol policies aimed at reducing alcohol‐related harms across the population (van der Velde et al., 2024). Evidence suggests these policies have successfully contributed to reductions in alcohol consumption, alcohol‐related diseases, and mortality (Babor et al., 2022; Månsson et al., 2024). However, both countries have recently taken steps toward relaxing these regulations. Sweden, for example, plans to introduce a new bill in 2025 that allows small‐scale alcohol producers to sell their products directly to visiting customers, bypassing the state‐owned alcohol monopoly Systembolaget. In Finland, the Alcohol Act of 2018 extended the serving hours for alcoholic beverages and permitted retail outlets other than the state monopoly to sell fermented alcoholic beverages containing up to 8% alcohol by volume. Åland, an autonomous region of Finland, has had a more relaxed alcohol policy for many years that preceded these national changes. As Åland lies outside the EU's VAT and excise duty area, ferries docking in the region can sell duty‐free alcohol on voyages between EU countries, which has resulted in greater accessibility of affordable alcoholic beverages for both residents and visitors (Karlsson & Österberg, 2009). Approximately 30% of the alcohol consumed by Åland's residents is purchased onboard these ferries (Karlsson, 1999).

## MATERIALS AND METHODS

### Sample and design

**FIGURE 1:**
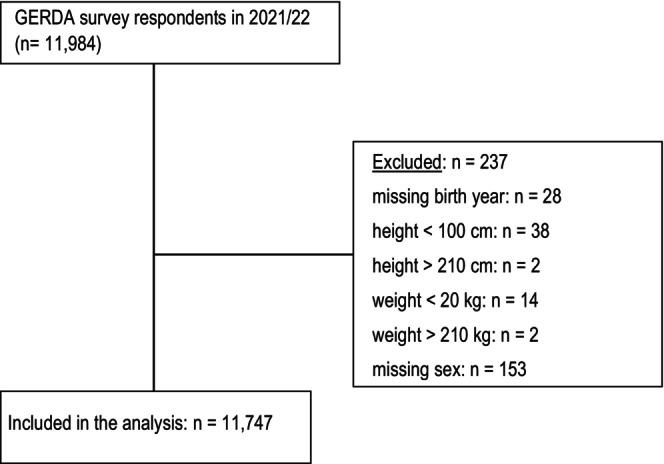
Study sample selection for the analysis.

Participants residing in the regions of Ostrobothnia, South Ostrobothnia, and the Åland Islands in Finland, as well as the Westrobothnia region in Sweden, responded to a postal survey conducted between late 2021 and early 2022 as part of the Gerontological Regional Database (GERDA) project. GERDA is a cross‐national, interdisciplinary collaboration focused on the health and psychosocial living conditions of older adults. The 2021/2022 survey followed similar sampling procedures as previous waves conducted in 2005, 2010, and 2016. A questionnaire was mailed to all individuals born in 1930, 1935, 1940, 1945, 1950, and 1955, with exceptions in the Swedish municipalities of Skellefteå and Umeå and the Finnish municipalities of Vaasa and Seinäjoki, where every second or third individual was contacted. The latest GERDA survey included additional items, such as the AUDIT‐C questions on alcohol consumption. The survey response rate in 2021/2022 was 59.1% in Sweden and 46.2% in Finland, with a total of 11,984 individuals responding. This study included 11,747 community‐dwelling older adults who responded to the 2021/2022 GERDA survey. Figure 1 illustrates the selection process for the study population.

The GERDA survey data collection received ethics approval from the Swedish Ethical Review Authority in 2021 (DNR: 2021‐04965). In Finland, the Medical Research Act 488/1999 exempts anonymous population‐based postal surveys from requiring ethical approval (see http://www.finlex.fi/en/laki/kaannokset/1999/en19990488).

### Measures

#### Hazardous and heavy episodic (binge) drinking

The primary outcome of interest in this study was alcohol use in the past 12 months, assessed using the Alcohol Use Disorders Identification Test‐Consumption (AUDIT‐C). The AUDIT‐C is a three‐item screening tool that yields scores ranging from 0 to 12 (Bush, 1998). It covers questions on drinking frequency (0 = never, 1 = monthly or less, 2 = 2–4 times a month, 3 = 2–3 times a week, or 4 = 4 or more times a week), usual quantity of alcohol (standard glasses) on a typical drinking day (0 = 1–2, 1 = 3–4, 2 = 5–6, 3 = 7–9, or 4 = 10 or more), and frequency of binge (heavy episodic) drinking, defined as consuming six or more drinks on one occasion (0 = never, 1 = less than monthly, 2 = monthly, 3 = weekly, 4 = daily or almost daily). In Sweden, one standard glass of alcohol corresponds to 12 grams of ethanol.

For the first outcome measure, scores were categorized as follows: *current abstinence* (AUDIT‐C = 0), *low‐risk alcohol use* (AUDIT‐C = 1–3), and *high‐risk/hazardous alcohol use* (AUDIT‐C ≥ 4), based on previously used thresholds for older adults (Aalto et al., 2011; Towers et al., 2011). The second outcome measure focused on HED. Participants who responded “monthly,” “weekly,” or “daily or almost daily” were categorized as *heavy episodic/binge drinkers*.

#### Covariates

##### Sociodemographic variables

Sociodemographic variables included *age* (categorized as 65, 70, 75, 80, 85, 90 years), *sex* (male/female), *education level* (primary or lower secondary, upper secondary, higher education; higher education defined as having enrolled in college or university‐level courses, regardless of graduation), *net income level* (1000 euros or less, between 1001 and 2000 euros, and above 2000 euros), *marital status* (single, divorced, and widow in one category, and married, and cohabiting in another category), and *region of residence* (Westrobothnia, Ostrobothnia, South Ostrobothnia, and the Åland Islands).

##### Other psychosocial variables

###### Depression

Depression was assessed using the four‐item Geriatric Depression Scale (GDS‐4) (D'Ath et al., 1994). McDonald's omega, a general estimator of reliability, which accounts for varying factor loadings, was 0.67 for GDS‐4. Participants scoring between two and four on the GDS‐4 were classified as having depressive symptoms. *Sleep quality* was assessed with the question “Do you have good sleep at night?” (yes/no). *Loneliness* was measured with the question “Do you suffer from loneliness?” (yes/no). *Life crisis* related to bereavement was measured by self‐reported death of close family members (children, partners or other relatives), in the past 12 months. *Religiosity* was measured by active membership to religious associations or parishes.

###### Inner strength

The 20‐item Inner Strength Scale (ISS) (Lundman et al., 2011) assessed inner strength. Each item was rated on a six‐point Likert scale (1 = “totally disagree” to 6 = “totally agree”), with total scores ranging from 20 to 120 (McDonald's omega = 0.92). Higher scores indicated greater inner strength. For analysis, ISS scores were centered at 20.

##### Functional status, health, and activity‐related variables

###### Cardiovascular diseases and other vascular disorders (cardiovascular diseases, for brevity)

Participants self‐reported diagnoses of stroke, heart attack, hypertension, or use of antihypertensive medications. *Polypharmacy* was defined as the regular use of five or more medications simultaneously (Masnoon et al., 2017).

###### Body mass index (BMI)

BMI was categorized into three groups: less than 23 kg/m^2^, between 23 and 29.9 kg/m^2^, and 30 kg/m^2^ or greater. These thresholds are specific to older adults and were derived from prior research (Winter et al., 2014).

###### Activities of daily living (ADL)

ADL independence was assessed using five binary items: one personal ADL (bathing) and four instrumental ADLs (shopping, food preparation, cleaning, and transportation) (Asberg & Sonn, 1989). McDonald's omega for the combined ADL measure was 0.74. Participants able to perform all tasks independently were considered ADL independent.

###### Frailty

Frailty was assessed using four items: reliance on mobility aids outdoors (reverse coded), feeling of exhaustion (reverse coded), ability to cross the road during green traffic lights, and ability to lift and carry heavy weights. The McDonald's omega value for this measure was 0.73. Participants who endorsed all items were classified as “Not frail.”

###### Physical activity

Physical activity levels were categorized according to the WHO recommended levels for older adults (WHO, 2010): participants reporting less than 150 min of moderate‐intensity physical activity per week were classified as having “low physical activity,” while those meeting or exceeding 150 min of moderate intensity or 75 min of vigorous‐intensity activity per week were classified as having “moderate physical activity.”

###### Subjective health/self‐rated health

It was assessed using the SF‐36 question (Ware & Sherbourne, 1992): “In general, would you say your health is: excellent, very good, good, fair, or poor?” Responses were trichotomized into “excellent/very good,” “good,” and “fair/poor” categories.

### Statistical analysis

All analyses were conducted using Stata version 18.5. Statistical significance was set at *p*‐values of <0.05. Multiple imputation by chained equations (MICE) was employed to impute, assuming data were missing at random. For variables consisting of multiple items, imputation was performed on individual items. Following the approach by Graham et al. (2007), the number of imputed datasets was set to 20. The percentages of missing values for the covariates ranged from 0.9% to 13.0%, with 84.2% of the imputed variables having a percentage of missing values less than 5.0%. Missing data for the three AUDIT‐C items were 3.4%, 4.0%, and 3.4% for the first, second, and third items, respectively.

The prevalences of hazardous and HED were estimated for men and women separately. Age categories were further grouped into two clusters: one including young‐olds aged 65, 70, and 75 years (i.e., born in the Years 1955, 1950, and 1945), and another including the oldest‐olds (aged 80, 85, and 90 years, i.e., born in the Years 1940, 1935, 1930) before estimating the prevalences of hazardous and HED in men and women by age group separately.

For all regression analyses, models were specified sequentially. Model 1 estimated bivariate associations between each independent variable and the outcome. Model 2 included sociodemographic factors, Model 3 added psychosocial variables, and Model 4 included physical health and functional status indicators in addition to variables from the previous models.

#### Hazardous alcohol use

Multinomial regression models assessed the association between drinking levels (current abstinence, lower risk, and hazardous drinking) and predictor variables, with a series of separate analyses conducted for men and women. Multinomial regression models were chosen over ordered logistic regression models due to Brant tests, which indicated that the proportional odds assumption was violated (Brant, 1990). For women, the regression models were based on mixed‐effects multinomial regression procedures, as a likelihood‐ratio test against standard multinomial regression suggested significant variability across the four regions. In contrast, the models for men were based on standard multinomial regression because of nonsignificant variability between the four regions. In all analyses, current abstinence and hazardous drinking were compared to lower risk drinking.

#### Heavy episodic drinking

Ordinary logistic regression models were used to examine the association between HED and predictor variables in men and women separately. Likelihood‐ratio tests suggested mixed‐effects logistic regression models did not provide a better fit compared to standard logistic regression models. For these analyses, current abstainers were included in the non‐HED group.

## RESULTS

### Prevalences of hazardous and heavy episodic drinking

**FIGURE 2:**
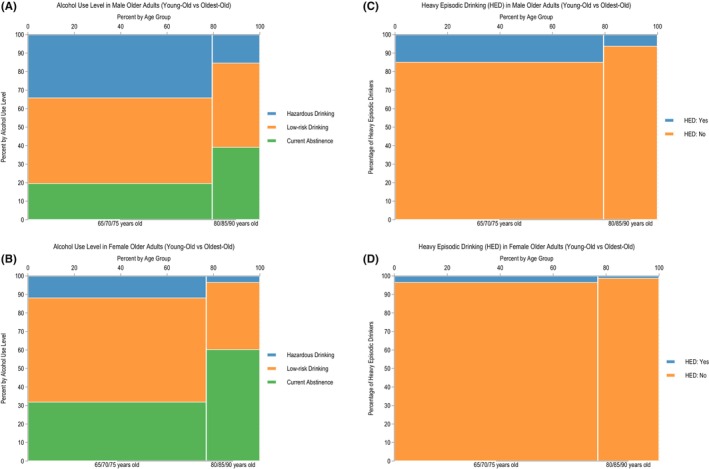
(A)–(D) Spine plots of hazardous and heavy episodic drinking by gender and age groups.

**FIGURE 3:**
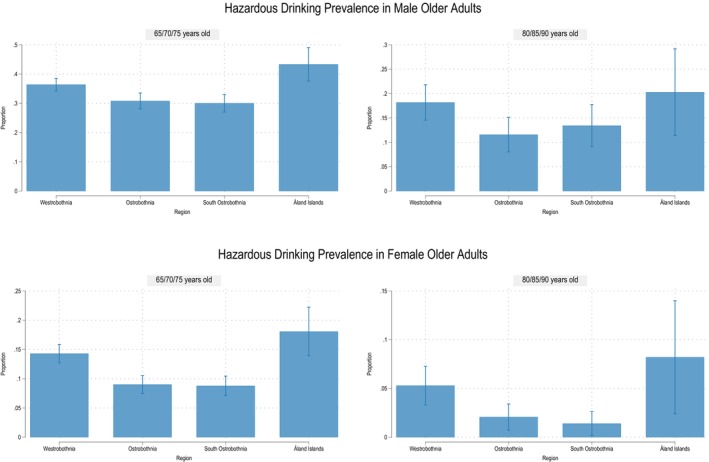
Prevalence of hazardous alcohol use by gender, age group, and region. Note the graphs are not on the same scale.

The sociodemographic characteristics of the study participants are detailed in Table 1, with supplementary descriptive statistics for all covariates provided in Table S1 (observed data) and Table S2 (imputed data). The overall prevalence of hazardous alcohol use, defined as an AUDIT‐C score of four or more points, was estimated to be 30.2% (95% CI, 29.0–31.4) among men and 9.8% (95% CI, 9.1–10.6) among women. The prevalence of hazardous alcohol use by sex and age group is presented in Figure 2A,B, as well as in Figure S1. Among participants aged 65, 70, or 75 years (young‐olds), the prevalence of hazardous drinking was 34.1% (95% CI, 32.6–35.5) in men and 11.7% (95% CI, 10.8–12.7) in women. For those aged 80, 85, or 90 years (oldest‐olds), the prevalence was lower, at 15.2% (95% CI, 13.1–17.4) in men and 3.4% (95% CI, 2.4–4.3) in women. Figure 3 presents the proportion of hazardous alcohol use for older men and women across the four regions. The Ostrobothnia and South Ostrobothnia regions had the lowest prevalence of hazardous drinking for both age groups and genders.

**TABLE 1:** Sociodemographic characteristics of study participants.

	Westrobothnia, *n* (%)	Ostrobothnia, *n* (%)	South Ostrobothnia, *n* (%)	Åland, *n* (%)	Total, *n* (%)	Missing, *n* (%)
Total	4890 (41.63)	3348 (28.50)	2693 (22.93)	816 (6.95)	11,747 (100.00)	0/11,747 (0.00)
Gender						
Men	2421 (49.51)	1489 (44.47)	1160 (43.07)	385 (47.18)	5455 (46.44)	0/11,747 (0.00)
Women	2469 (50.49)	1859 (55.53)	1533 (56.93)	431 (52.82)	6292 (53.56)	
Age group						
65	1959 (40.06)	767 (22.91)	736 (27.33)	232 (28.43)	3694 (31.45)	0/11,747 (0.00)
70	962 (19.67)	884 (26.40)	723 (26.85)	203 (24.88)	2772 (23.60)	
75	1025 (20.96)	884 (26.40)	586 (21.76)	208 (25.49)	2703 (23.01)	
80	500 (10.22)	401 (11.98)	314 (11.66)	112 (13.73)	1327 (11.30)	
85	329 (6.73)	281 (8.39)	223 (8.28)	50 (6.13)	883 (7.52)	
90	115 (2.35)	131 (3.91)	111 (4.12)	11 (1.35)	368 (3.13)	
Marital status						
Single/divorced/widow	1457 (30.20)	911 (27.27)	911 (27.27)	239 (29.73)	3444 (29.59)	108/11,747 (0.92)
Married/partner	3367 (69.80)	2430 (72.73)	2430 (72.73)	565 (70.27)	8195 (70.41)	
Education level						
Primary/lower secondary	1621 (33.63)	1305 (39.17)	920 (34.30)	184 (22.63)	4030 (34.60)	100/11,747 (0.85)
Upper secondary	1774 (36.80)	1625 (48.77)	1492 (55.63)	460 (56.58)	5351 (45.94)	
Higher education	1425 (29.56)	402 (12.06)	270 (10.07)	169 (20.79)	2266 (19.46)	
Income after tax						
≤1000 €	615 (12.82)	480 (14.83)	439 (16.72)	74 (9.40)	1608 (14.04)	298/11,747 (2.54)
1001–2000 €	3460 (72.10)	2303 (71.15)	1882 (71.67)	454 (57.69)	8099 (70.74)	
>2000 €	724 (15.09)	454 (14.03)	305 (11.61)	259 (32.91)	1742 (15.22)	

**FIGURE 4:**
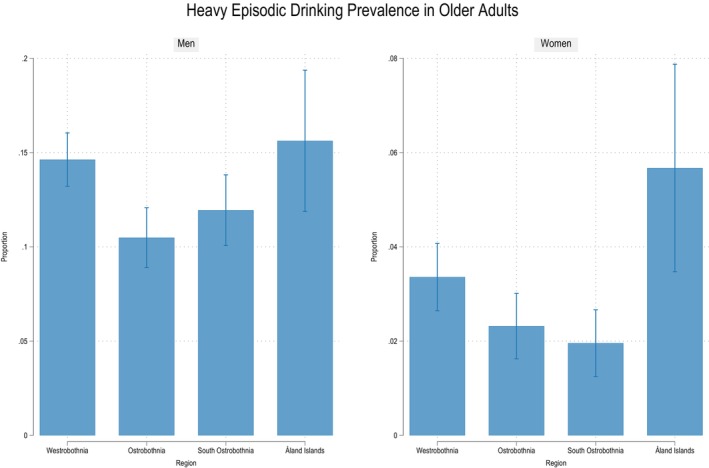
Prevalence of heavy episodic drinking by gender and region. Note the graphs are not on the same scale.

The prevalence of HED was estimated at 13.0% (95% CI, 12.1–13.9) in men and 2.9% (95% CI, 2.5–3.3) in women. In the young‐old cohort, HED prevalence was 14.8% (95% CI, 13.7–15.8) among men and 3.4% (95% CI, 2.9–3.9) among women. In the oldest‐old cohort, the prevalence was 6.1% (95% CI, 4.7–7.5) in men and 1.2% (95% CI, 0.6–1.7) in women (Figure 2C,D). The regional patterns of HED prevalence for both men and women mirrored those of hazardous alcohol use, with the Åland Islands showing the highest prevalence and the Ostrobothnia and South Ostrobothnia regions showing the lowest (Figure 4).

### Correlates of hazardous drinking

#### Older men

**FIGURE 5:**
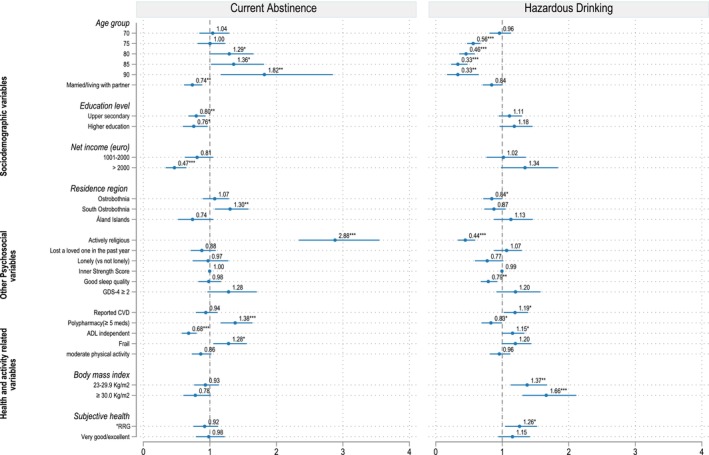
Correlates of hazardous alcohol use in male older adults. Reference outcome = low‐risk drinking. *Reference categories for*: Age group = 65; Marital status = single/divorced/widow; Education level = primary or lower secondary level; Net income = ≤1000 euro; Residence region = Westrobothnia; Religiousness = no/passive; Loss of loved ones = none; Sleep quality = bad; Depression = GDS < 2; Reported CVD = none; Polypharmacy = <5 medications; ADL = not fully independent; Frailty = not frail; Physical activity = <150 min/week; BMI = <23 kg/m^2^; Subjective health = fair/bad. Statistical significance is denoted as *p < 0.05, **p < 0.01, ***p < 0.001.

Figure 5 presents the relative risk ratios (RRRs) from the fully adjusted standard multinomial regression model for men, which compares current abstinence and hazardous drinking to low‐risk drinking. The corresponding RRRs, 95% confidence intervals (CIs), and *p*‐values for all variables included in the models are summarized in Table S3, which also presents results from all four sequential models, including bivariate associations from unadjusted models.

Among men, the likelihood of current abstinence compared to low‐risk drinking increased with age, especially among the oldest‐olds, aged 80, 85, and 90 years, who were more likely to abstain from alcohol than those aged 65 years (Figure 5). Men who were actively practicing religion, those taking multiple medications (polypharmacy), and those classified as frail were more likely to be abstinent than to engage in low‐risk drinking. Men from South Ostrobothnia were also more likely to abstain than those from Westrobothnia. In contrast, men who were married or living with a partner, had upper secondary or higher level education, earned more than 2000 euros per month, and were fully independent in activities of daily living (ADL) were less likely to abstain from alcohol and more likely to engage in low‐risk drinking.

The probability of hazardous drinking versus low‐risk drinking was higher among older men living with cardiovascular diseases (RRR = 1.19; 95% CI, 1.02–1.39), those fully independent in ADL (RRR = 1.15; 95% CI, 1.00–1.33), and those who rated their overall health as good (RRR = 1.26; 95% CI, 1.04–1.52). Compared to men with a BMI less than 23 kg/m^2^, those with a BMI between 23 and 29.9 kg/m^2^ (RRR = 1.37; 95% CI, 1.13–1.67) and a BMI of 30 kg/m^2^ or more (RRR = 1.66; 95% CI, 1.31–2.11) were more likely to engage in hazardous drinking compared to low‐risk drinking (Figure 5, Table S3).

In contrast, compared to men aged 65 years, the probability of hazardous drinking versus low‐risk drinking was lower for those aged 75 years (RRR = 0.56; 95% CI, 0.47–0.67), 80 years (RRR = 0.46; 95% CI, 0.35–0.59), 85 years (RRR = 0.33; 95% CI, 0.23–0.48), and 90 years (RRR = 0.33; 95% CI, 0.17–0.65). Men who were actively practicing religion (RRR = 0.44; 95% CI, 0.33–0.59), those reporting good sleep quality (RRR = 0.79; 95% CI, 0.68–0.92), and those with polypharmacy (RRR = 0.83; 95% CI, 0.69–1.00) were also less likely to drink at high‐risk level than engaging in low‐risk drinking. Additionally, men from Ostrobothnia were less likely to engage in hazardous drinking than those from Westrobothnia.

#### Older women

**FIGURE 6:**
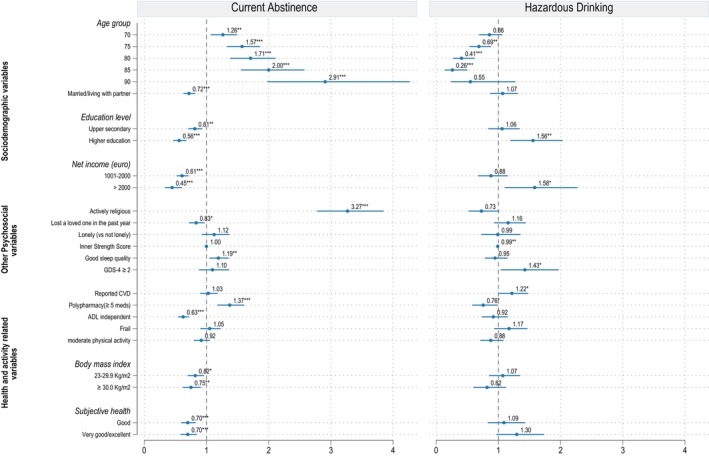
Correlates of hazardous alcohol use in female older adults. Reference outcome = low‐risk drinking. Reference categories for: Age group = 65; Marital status = single/divorced/widow; Education level = primary or lower secondary level; Net income = ≤1000 euro; Religiousness = no/passive; Loss of loved ones = none; Sleep quality = bad; Depression = GDS < 2; Reported CVD = none; Polypharmacy = <5 medications; ADL = not fully independent; Frailty = not frail; Physical activity = <150 min/week; BMI = <23 kg/m^2^; Subjective health = fair/bad. Statistical significance is denoted as *p < 0.05, **p < 0.01, ***p < 0.001.

Figure 6 displays the relative risk ratios (RRRs) from the fully adjusted mixed‐effect multinomial regression model for women, comparing current abstinence and hazardous drinking to low‐risk drinking. Table S4 provides a detailed summary of RRRs, 95% confidence intervals (CIs), and exact *p*‐values for all variables in the four sequential models.

The estimated variance of the random effect at region level was 0.03 (95% CI, 0.01–0.17), corresponding to a standard deviation of 0.18, which implies that for every one standard deviation change, the RRR changes by a factor of exp. (0.18) or 1.20 (95% CI, 1.08–1.52). After accounting for this regional variance in women, the likelihood of current abstinence compared to low‐risk drinking significantly increased with age. Similar to the findings in men, active religious practice and polypharmacy were positively associated with abstinence compared to low‐risk drinking (Figure 6). Women reporting good sleep quality were also more likely to avoid drinking than drink at lower level. Conversely, a negative association with abstinence (versus low‐risk drinking) was observed among women who were married or living with a partner, had upper secondary or higher level education, had higher income, higher BMI, were fully independent in ADL, and those who rated their overall health as good or excellent.

The probability of hazardous drinking versus low‐risk drinking was greater among women with higher education (RRR = 1.56; 95% CI, 1.19–2.03), a monthly income of 2000 euros (RRR = 1.58; 95% CI, 1.10–2.27), those living with depressive symptoms (RRR = 1.43; 95% CI, 1.04–1.97), and those with cardiovascular diseases (RRR = 1.22; 95% CI, 1.00–1.48) (Figure 6, Table S4). On the other hand, compared to women aged 65 years, the probability of hazardous drinking versus low‐risk drinking was lower for those aged 75 years (RRR = 0.69; 95% CI, 0.53–0.88), 80 years (RRR = 0.41; 95% CI, 0.27–0.62), and 85 years (RRR = 0.26; 95% CI, 0.14–0.50). Additionally, women with higher ISS scores (RRR = 0.99; 95% CI, 0.98–1.00), and those with polypharmacy (RRR = 0.76; 95% CI, 0.58–0.99) were less likely to engage in hazardous drinking compared to low‐risk drinking.

### Correlates of heavy episodic drinking

**FIGURE 7:**
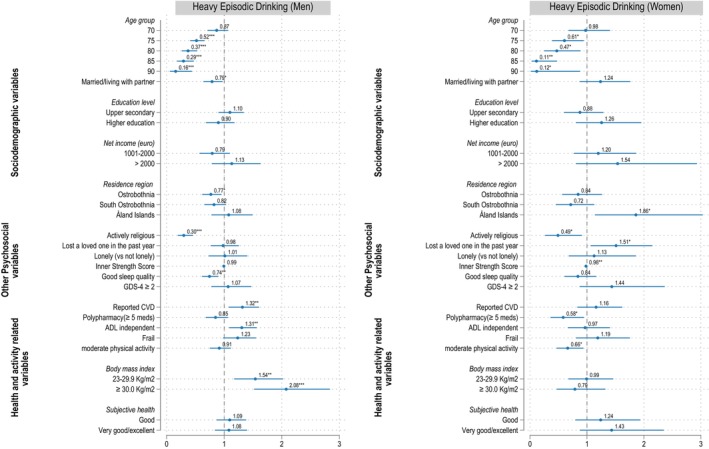
Correlates of heavy episodic drinking in men and women. Reference categories for: Age group = 65; Marital status = single/divorced/widow; Education level = Primary or lower secondary level; Net income = ≤1000 euro; Residence region = Westrobothnia; Religiousness = no/passive; Loss of loved ones = none; Sleep quality = bad; Depression = GDS < 2; Reported CVD = none; Polypharmacy = <5 medications; ADL = not fully independent; Frailty = not frail; Physical activity = <150 min/week; BMI = <23 kg/m^2^; Subjective health = fair/bad. Statistical significance is denoted as *p < 0.05, **p < 0.01, ***p < 0.001.

Figure 7 illustrates the adjusted odd ratios (ORs) for HED versus the abstainer/non‐HED group, based on fully adjusted ordinary logistic regression models for men and women. The corresponding ORs, 95% confidence intervals (CIs), and exact *p*‐values for all variables included in the models, including bivariate associations from unadjusted models, are summarized in Table S5 for men and Table S6 for women. Overall, the associations between predictor variables and HED mirrored the trends observed for hazardous drinking in the multinomial models.

For men, older age, being married or living with a partner, residing in Ostrobothnia, active religious practice, and good sleep quality were all associated with a reduced likelihood of HED. In contrast, men with cardiovascular diseases, higher BMI, and full independence in activities of daily living (ADL) were more likely to engage in HED than abstain or drink fewer drinks per drinking occasion.

For women, older age and active participation in religious activities similarly reduced the likelihood of HED. Additionally, higher scores on the inner strength scale, polypharmacy, and greater levels of physical activity were associated with a decreased likelihood of HED. On the other hand, women residing in the Åland Islands and those who had recently lost a close family member had a higher likelihood of HED.

## DISCUSSION

This cross‐sectional study, using data from the 2021/2022 GERDA survey, examined the prevalence and the correlates of hazardous and HED among Swedish and Finnish older adults born in between 1930 and 1955. We found that even if there are varying patterns of alcohol use across age groups, gender, and regions, the proportions of older adults who engage in hazardous and heavy episodic drinking were elevated, with notably higher prevalence among men in the younger cohort. Moreover, both hazardous and heavy episodic drinking were associated with a range of sociodemographic, psychological, and health‐related factors, albeit with some gender‐specific variation.

### Gender, age, and contextual differences in drinking patterns

In the present study, approximately 30% of older men and 10% of older women were hazardous drinkers, and similarly, HED was more common among men (13.0%) than women (2.9%), with younger‐old participants generally drinking at higher risk levels than the oldest‐old. These findings align with recent research from Nordic and other Western countries, which has documented elevated levels of problematic alcohol use among older adults: 34% in Swedish adults aged 65–84 years (Ramstedt & Guttormsson, 2024), between 23% in Portugal and 42% in Denmark among adults aged 60–75 years across four European countries (Rossow & Træen, 2020), and 40% in Norwegian adults aged 60–99 years (Stelander et al., 2022). Additionally, our study observed regional variations in drinking patterns with lower prevalence of hazardous and heavy episodic drinking in Ostrobothnia and South Ostrobothnia. The 2022 Healthy Finland Survey conducted by the Finnish Institute for Health and Welfare (THL) similarly reported lower levels of hazardous drinking among older adults in these regions compared to other parts of Finland, including Åland (THL, 2022a). This trend may be explained by historical cultural and religious factors, as these regions have historically been centers of the temperance movement and are often considered parts of Finland's Bible Belt (Smith, 2017).

The lower prevalence of hazardous and heavy episodic drinking and the higher gender gap observed among those aged 80, 85, and 90 years (i.e., very old adults) relative to older adults aged 65, 70, and 75 years may reflect several factors. First, alcohol use tends to decline with advancing age due to physiological changes and increasing disease burden, especially among older cohorts (Moore et al., 2005). Second, evidence from Nordic and other Western countries suggests that those born in the post–World War II era (the so‐called baby boom generation), who came of age in a more liberal alcohol culture, tend to drink alcohol more frequently and at higher levels compared to earlier cohorts (Ahlner et al., 2018; Bryazka et al., 2022). While some of these cohort effects are observed across both genders, the most marked changes have occurred in women's drinking patterns, resulting in a narrowing of the gender gap in drinking, with increasing alcohol consumption observed among women (Grucza et al., 2018; Slade et al., 2016).

Nonetheless, it is important to state that the operationalization of hazardous drinking and HED varies across studies and national guidelines. While some investigations apply different AUDIT‐C cutoffs (Stelander et al., 2022; Trias‐Llimós et al., 2022), others employ different criteria such as total weekly alcohol consumption, national guidelines, or WHO risk drinking levels (Ahlner et al., 2018; Kuerbis, 2020; Rossow & Træen, 2020). In this study, we define hazardous drinking as an AUDIT‐C score of four or more (Aalto et al., 2011; Towers et al., 2011) and HED, based on the third item of AUDIT‐C, as consuming 6+ standard glasses of alcohol on one occasion at least once per month (Towers et al., 2011). To note, the Finnish Institute for Health and Welfare (THL) applies a similar threshold for hazardous drinking among adults aged 65 years or older as the one used in this study, but it categorizes HED as occurring at least once per week rather than per month, which is a more stringent criterion and would reduce the observed prevalence of HED if applied here (THL, 2022b). In contrast, the Swedish National Board of Health and Welfare (NBHW) recently updated its recommendations for healthcare interventions targeting at‐risk drinking (NBHW, 2024). These guidelines define at‐risk drinking as 10 or more standard drinks per week, and HED as four or more standard drinks on one occasion at least once per month, without age‐specific distinctions. If we had employed the NBHW's definition of HED (4+ drinks rather than 6+), we might have captured an even larger segment of older adults as engaging in intensive drinking.

### Sociodemographic and psychosocial correlates of hazardous and heavy episodic drinking

Other social factors were also associated with drinking levels in this study. Being married or living with a partner lowered the odds of past 12‐month abstinence (versus lower risk drinking) in both men and women. However, no significant association was observed between marital status and hazardous drinking in either gender. Although initial models for older men suggested a protective association between having a partner and hazardous drinking, this relationship lost significance after introducing health and activity‐related variables into the final model. These findings are consistent with previous studies, which have likewise reported no protective association between marriage and hazardous alcohol use among older adults (Ahlner et al., 2022; Immonen et al., 2011b; Rossow & Træen, 2020; Stelander et al., 2022). Our observation that married or cohabiting older men but not women have lower odds of heavy episodic drinking aligns with studies reporting reduced likelihoods of binge drinking in men but not women (Blazer & Wu, 2009; Stelander et al., 2022). By contrast, some investigations have reported no association between marital status and heavy episodic drinking in older populations (Han et al., 2019; Towers et al., 2011); however, these studies did not conduct gender‐stratified analyses. Even if the cross‐sectional nature of the data did not allow us to investigate the association between alcohol use and marital status at different time points, previous longitudinal studies suggest men, but not women, may be more likely to engage in binge drinking as a coping mechanism following marital dissolution (Reczek et al., 2016; Williams & Dunne‐Bryant, 2006). Moreover, research on alcohol and marital dynamics indicates that partners' drinking behaviors may converge over time via social control, where one partner, often the wife, regulates the other's heavy drinking, or through social contagion, in which one partner's consumption level shifts to match the other's (Reczek et al., 2016).

In this study, older adults with greater socioeconomic advantage were less likely to abstain than drink, and this was particularly evident among women with university‐level education and higher incomes, who had an increased likelihood of hazardous drinking. These findings align with previous research reporting a positive association between higher socioeconomic status and hazardous drinking in older adults (Ahlner et al., 2022; Immonen et al., 2011b; Moore et al., 2005; Stelander et al., 2022). Studies with gender‐stratified analyses, however, have produced inconsistent findings regarding the relationship between education level and problematic alcohol use. While our results are in line with previous studies suggesting a positive association between higher education level and hazardous drinking in women but not men (Blazer & Wu, 2009; Trias‐Llimós et al., 2022), other investigations have reported similar positive associations in older men (Stelander et al., 2022; Tschorn et al., 2023). Despite these discrepancies, the link between higher education and increased alcohol use may reflect a more liberal attitude toward alcohol among current older adults, especially highly educated women with greater social and financial freedoms. Indeed, many older adults perceive alcohol use as an indicator of a good quality of life and a component of healthy aging (Jemberie et al., 2023).

Previous studies have also reported that while nonabstinence is generally more common in higher socioeconomic groups, individuals with socioeconomic disadvantage are more likely to engage in heavy episodic drinking (Grittner et al., 2013) and may be more vulnerable to alcohol‐related harms, including mortality (Probst et al., 2020) due to factors such as cumulative disadvantage, comorbidities, and limited access to healthcare (Bellis et al., 2016). As a result, we do not rule out that the associations between socioeconomic status and alcohol use observed in this study may be affected by the possibility that individuals from lower socioeconomic backgrounds with problematic alcohol use did not survive to older age, and that those who did may have ceased drinking due to illness.

Active participation in religious groups or parishes was consistently associated with a lower likelihood of engaging in hazardous and heavy episodic drinking and a higher likelihood of current abstinence among both older men and women. Previous studies indicate that regular attendance at religious services and involvement in religious communities often correlates with healthier behaviors, including reduced alcohol consumption (Ahlner et al., 2022; Demir‐Dagdas & Child, 2019). Religious communities can provide social support and establish norms that discourage excessive alcohol use, while religious beliefs and practices may help individuals maintain a sense of purpose and cope with health and psychosocial crises (Ellison & Levin, 1998).

In contrast, our findings suggest other psychosocial factors differentially affect the odds of hazardous and heavy episodic drinking by gender, with stronger association observed among older women. Although bereavement and loneliness have previously been linked to problematic alcohol use in older adults (Kuerbis, 2020), in our study, only bereavement in older women was associated with a greater risk of binge drinking. Likewise, higher scores on the inner strength scale (a measure of inner resources that contribute to resilience and self‐efficacy, particularly in the face of stress, adversity, or illness) were associated with lower likelihoods of hazardous and heavy episodic drinking only in older women. Inner strength has previously been linked to improved well‐being and reduced odds of depression (Boman et al., 2015; Viglund et al., 2022).

Further underscoring these gender‐specific patterns, depression in our study was associated with hazardous drinking only among older women. Studies on clinical samples of older adults report a high prevalence of co‐occurring depression and alcohol use disorder, especially among women (Bartels et al., 2006; Jemberie et al., 2022). However, research on community‐dwelling older populations presents mixed conclusions on the link between depression and hazardous drinking (Ahlner et al., 2022; Hajat et al., 2004; Smith & Shevlin, 2008) although some older adults report alleviating depressive symptoms as a reason for heavy drinking (Immonen et al., 2011a). Given the positive associations of bereavement and depression with problematic alcohol use and the negative association of inner strength with such use, it is plausible that some older women may turn to hazardous or heavy episodic drinking to cope with psychological stressors.

Finally, our study suggests that poor sleep was associated with higher odds of both hazardous and heavy episodic drinking in older men, whereas in older women it was linked only to a reduced likelihood of abstinence (i.e., they were more likely to drink at lower risk levels rather than abstain). While we cannot establish temporality in a cross‐sectional design, results from earlier studies suggest a bidirectional association between sleep disturbances and problematic alcohol use (Chakravorty et al., 2016). For example, previous research suggests some older adults with insomnia use alcohol to facilitate sleep (Haighton et al., 2018) due to its effect on reducing sleep latency. On the other hand, several sleep‐related problems have been reported among older adults with hazardous drinking (Hussain et al., 2022; Zheng et al., 2024), even if evidence regarding gender differences remains mixed.

### Functional status and health‐related correlates of hazardous and heavy episodic drinking

The present study found that, compared to lower risk drinking, the likelihood of current abstinence increased with age, while the likelihood of hazardous and heavy episodic drinking decreased with advancing age in both older men and women. Previous research indicates that as health declines and chronic illnesses accumulate, many older adults reduce or stop drinking altogether (Dawson et al., 2013; Holdsworth et al., 2016). Our findings suggest that indicators of multimorbidity and diminished functional status (e.g., polypharmacy, lower independence in daily life) were generally associated with current abstinence or lower risk drinking rather than hazardous or heavy episodic drinking. Since this study did not distinguish former drinkers from lifetime abstainers, some abstainers may be “sick quitters,” individuals who stopped drinking due to severe health problems.

Perceiving one's health as good or excellent was associated with continued drinking rather than abstention in our study. Older men who rated their health as good were more likely to engage in hazardous drinking, while older women who viewed their health as good to excellent were more likely to report lower risk drinking rather than abstaining. Many older adults believe alcohol can have positive health effects (Bareham et al., 2019) and may use it as self‐medication (Immonen et al., 2011a). Some also regard alcohol as part of healthy aging, and a perceived lack of threat from alcohol to physical and mental health can lower the cognitive appraisal of alcohol problems and delay treatment seeking (Jemberie et al., 2023).

Our findings further indicate that older women with higher body mass index (BMI) were more likely to engage in lower risk drinking than to abstain, while older men with higher BMI were more likely to drink at a hazardous level. Additionally, both men and women who reported any cardiovascular or vascular disease (CVD) were more likely to engage in hazardous drinking than to drink moderately, and older men with CVD were also more likely to report heavy episodic drinking. Although some participants reported conditions such as myocardial infarction or stroke, hypertension was the most common vascular problem. Despite their diagnosis, most older adults with CVD rated their health as good or excellent, which may in part explain their continued engagement in higher risk drinking behaviors. Hazardous alcohol use has been previously linked to hypertension and a sedentary lifestyle among older adults (Steptoe & McMunn, 2009), and a reduction in alcohol consumption in people with hazardous drinking is associated with a significant blood pressure reduction (Roerecke et al., 2017). Nevertheless, despite the positive association between hazardous drinking and CVD risk, many older adults are exposed to inaccurate health information on the effects of alcohol on cardiovascular health, which can increase their odds of hazardous drinking (Welfordsson et al., 2024).

The study findings should be interpreted with caution. First, the cross‐sectional design precludes drawing causal inferences from the reported results. Second, the observed differences in hazardous and heavy episodic drinking between age groups may reflect not only age effects but also period or cohort effects, which cannot be disentangled with data from a single wave. Third, the analysis was based on unweighted data. Nevertheless, prevalences reported are consistent with previous population studies on problematic alcohol use among older adults in Sweden and Finland as previously discussed. Fourth, the use of self‐reported data introduces a greater risk of response bias, potentially stemming from recall inaccuracies or social desirability (Althubaiti, 2016). This can result in underreporting of undesirable health behaviors, such as heavy drinking. Fifth, the analyses did not adjust for smoking due to lack of data. Based on estimates from The Tobacco Atlas (tobaccoatlas.org/factsheets/), adult smoking prevalence is 12.3% in Sweden and 15% in Finland. Finally, despite the respectable response rate, older adults with severe functional limitations or alcohol use disorder may be underrepresented in the survey, potentially affecting the associations observed in this study.

Despite these limitations, the study has several strengths. The 2021–2022 cross‐national Gerontological Regional Database (GERDA) survey data includes participants from six birth cohorts, three of which represent the oldest‐old group aged 80, 85, and 90 years. The use of multiple validated instruments allowed for the inclusion of a wide range of biopsychosocial variables. Additionally, the analysis addressed missing data through multiple imputation, reducing potential biases due to incomplete responses. The analytical approach also accounted for regional differences, which provided more robust estimates of the relationships between individual variables and drinking behaviors.

## CONCLUSIONS

Problematic alcohol use is prevalent among older adults in Sweden and Finland, with some variations across regions and age groups. Gender differences are observed in the relationship between problematic alcohol use and demographic, psychosocial, functional, and health‐related factors. Problematic alcohol use in women is associated with higher socioeconomic status and reduced psychosocial resources related to bereavement, depression, and inner strength. Meanwhile, functional and health‐related factors are significant predictors of problematic alcohol use in men. Across both genders, religious participation serves as a protective factor, while self‐reported cardiovascular disease is associated with increased risk of hazardous drinking. These findings suggest the need for interventions that focus on strengthening resilience to psychosocial stressors and providing clear health communication about alcohol's harmful effects on cardiovascular and overall health. Given the increasing longevity and changing drinking patterns among older adults, healthcare providers could integrate routine alcohol screening and brief interventions that consider different biopsychosocial risk factors. Future longitudinal research is needed to better understand the temporal relationships between these factors and problematic alcohol use in later life.

## AUTHOR CONTRIBUTIONS

All authors contributed substantially to the manuscript. All authors reviewed and approved submission of the manuscript to *Alcohol, Clinical and Experimental Research*. Wossenseged Birhane Jemberie: Conceptualization, methodology, formal analysis, investigation, writing—original draft, writing—review & editing. Johan Niklasson: Conceptualization, writing—review & editing, validation. Knut Lönnroth: Conceptualization, writing—review & editing, validation. Erika Boman: Conceptualization, formal analysis, writing—review & editing, validation.

## FUNDING INFORMATION

An award from The Swedish Research Council for Health, Working Life and Welfare (FORTE, Grant no. 2019‐01453) supported WBJ's postdoctoral fellowship. An award from the Waldemar von Frenckell Foundation supported EB's contribution to the manuscript. The funders were not involved in the design, analysis and writing of the study or decision to submit manuscripts for publication. The 2021/2022 GERDA survey was supported by: Svensk‐Österbottniska Samfundet, Högskolestiftelsen i Österbotten, Vaasa Aktia Foundation, the Swedish Cultural Foundation, the Regional Council of South Ostrobothnia, Ålands självstyrelses 75‐års jubileumsfond and the Government of Åland.

## CONFLICT OF INTEREST STATEMENT

The authors declare that the research was conducted without any commercial or financial relationships that could be construed as a potential conflict of interest.

## ETHICS STATEMENT

The GERDA project received ethical approval from the Swedish Ethical Review Authority in 2021 (DNR: 2021‐04965). In Finland, the Medical Research Act 488/1999 exempts anonymous population‐based postal surveys from requiring ethical approval (see http://www.finlex.fi/en/laki/kaannokset/1999/en19990488). The study was conducted in accordance with the Helsinki Declaration and the Belmont Report. All data was collected and processed in accordance with the General Data Protection Regulation (GDPR). Participants were not provided compensation for study participation.

## Supporting information


Appendix S1

## Data Availability

The dataset presented in this study is not publicly available because of legal and ethical restrictions. Codes for data preparation and analyses, and Stata log files can be shared through correspondence with WBJ (wossenseged.jemberie@umu.se). Access to the data can be granted through an application to the GERDA steering group. Contact details and more information can be found on the website: www.gerdacenter.com.
