# Supplementary material for: Prevalence and gender‐specific correlates of hazardous and binge drinking among Swedish and Finnish older adults
Source: Alcohol Clin Exp Res (Hoboken). 2025 Aug 1;49(8):1744–58. doi: 10.1111/acer.70098 (PMC12365579; doi:10.1111/acer.70098)
Supplement: Supplementary file 1 — Appendix S1 [file ACER-49-1744-s001.docx]

**PREVALENCE AND GENDER-SPECIFIC CORRELATES OF HAZARDOUS AND BINGE DRINKING AMONG SWEDISH AND FINNISH OLDER ADULTS**

**Authors’ List, Order and Affiliation**

**Wossenseged Birhane Jemberie, MPH, PhD. ^1, 2*^**

Postdoctoral Fellow. **1.** Department of Social Work, Umeå University, 901 87 Umeå, Sweden;

**2.** Centre for Demography and Aging Research (CEDAR), Umeå University, Umeå, Sweden;

E-mail: [wossenseged.jemberie@umu.se](mailto:wossenseged.jemberie@umu.se) ORCiD: 0000-0002-4378-6803

**Johan Niklasson, MD, PhD. ^3^**

Associate professor of geriatrics, senior consultant (attending) physician. **3.** Department of Community Medicine and Rehabilitation, Geriatric Medicine, Sunderby Research Unit, Umeå University, Umeå, Sweden;

Email: [johan.niklasson@umu.se](mailto:johan.niklasson@umu.se) ORCiD: 0000-0001-5050-3720

**Knut Lönnroth, MD, PhD. ^4, 5^**

Professor of social medicine. **4.** Department of Global Public Health, Karolinska Institutet, Stockholm, Sweden; **5**. Chief Medical Officer, Government of Åland, Åland;

Email: [knut.lonnroth@regeringen.ax](mailto:knut.lonnroth@regeringen.ax) ORCiD: 0000-0001-5054-8240

**Erika Boman, PhD, RNT. ^6, 7^**

Associate professor of nursing. **6**. Department of Nursing, Umeå University, Umeå, Sweden; **7**. Research Affiliate. Åland University of Applied Sciences, Åland;

Email [erika.boman@ha.ax](mailto:erika.boman@ha.ax) ORCiD: 0000-0002-3989-609X

***Send correspondence to** Wossenseged Birhane Jemberie. Department of Social Work, Umeå University, 901 87 Umeå, Sweden. E-mail: [wossenseged.jemberie@umu.se](mailto:wossenseged.jemberie@umu.se)

**Running head: *Hazardous and Binge Drinking in Older Adults***

**Supplementary Materials**

**Supplementary Figure S1**. Prevalence of hazardous alcohol use (left column) and heavy episodic drinking (right column) by gender and age groups

**Supplementary Table S1.** Descriptive statistics for all covariates - observed data.

**Supplementary Table S2**. Descriptive statistics for all covariates – imputed data (imputations = 20)**.**

**Supplementary Table S3.** RRRs, 95% confidence intervals (CIs), and p-values from standard multinomial regression models comparing current abstinence and hazardous drinking to low-risk drinking among men.

**Supplementary Table S4.** RRRs. 95% confidence intervals (CIs), and p-values from mixed-effects multinomial regression models comparing current abstinence and hazardous drinking to low-risk drinking among women.

**Supplementary Table S5.** ORs, 95% confidence intervals (CIs), and p-values from ordinary logistic regression models for heavy episodic drinking among men.

**Supplementary Table S6.** ORs, 95% confidence intervals (CIs), and p-values from ordinary logistic regression models for heavy episodic drinking among women.

# **Supplementary Figure S1**. Prevalence of hazardous alcohol use (left column) and heavy episodic drinking (right column) by gender and age groups

**
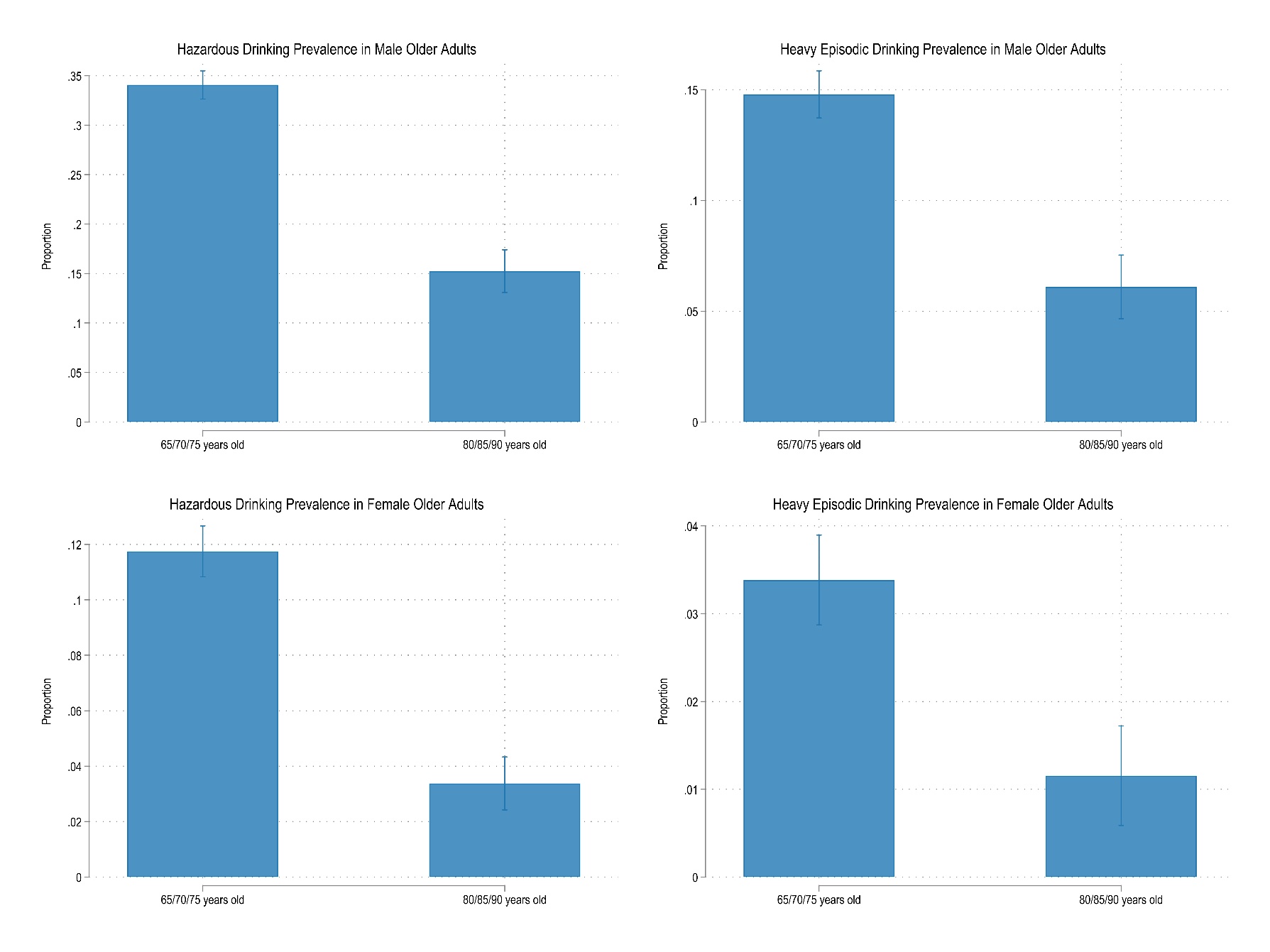
**

# **Supplementary Table S1.** Descriptive statistics for all covariates - observed data.

|  | **Westrobothnia**  **n (%)** | **Ostrobothnia**  **n (%)** | **South Ostrobothnia**  **n (%)** | **Åland**  **n (%)** | **Total**  **n (%)** | **Missing**  **n (%)** |
| --- | --- | --- | --- | --- | --- | --- |
| **Total** | 4890 (41.63) | 3348 (28.50) | 2693 (22.93) | 816 (6.95) | 11747 (100.00) | 0/11747 (0.00) |
| **Gender**  Men  Women | 2421 (49.51)  2469 (50.49) | 1489 (44.47)  1859 (55.53) | 1160 (43.07)  1533 (56.93) | 385 (47.18)  431 (52.82) | 5455 (46.44)  6292 (53.56) | 0 / 11747 (0.00) |
| **Age group**  65  70  75  80  85  90 | 1959 (40.06)  962 (19.67)  1025 (20.96)  500 (10.22)  329 (6.73)  115 (2.35) | 767 (22.91)  884 (26.40)  884 (26.40)  401 (11.98)  281 (8.39)  131 (3.91) | 736 (27.33)  723 (26.85)  586 (21.76)  314 (11.66)  223 (8.28)  111 (4.12) | 232 (28.43)  203 (24.88)  208 (25.49)  112 (13.73)  50 (6.13)  11 (1.35) | 3694 (31.45)  2772 (23.60)  2703 (23.01)  1327 (11.30)  883 (7.52)  368 (3.13) | 0 / 11747 (0.00) |
| **Marital status**  Single/divorced/widow  Married/partner | 1457 (30.20)  3367 (69.80) | 911 (27.27)  2430 (72.73) | 911 (27.27)  2430 (72.73) | 239 (29.73)  565 (70.27) | 3444 (29.59)  8195 (70.41) | 108 / 11747 (0.92) |
| **Education level**  Primary/ lower 2ndary  Upper secondary  Higher education | 1621 (33.63)  1774 (36.80)  1425 (29.56) | 1305 (39.17)  1625 (48.77)  402 (12.06) | 920 (34.30)  1492 (55.63)  270 (10.07) | 184 (22.63)  460 (56.58)  169 (20.79) | 4030 (34.60)  5351 (45.94)  2266 (19.46) | 100 / 11747 (0.85) |
| **Income after tax**  ≤1000 €  1001-2000 €  ˃2000 € | 615 (12.82)  3460 (72.10)  724 (15.09) | 480 (14.83)  2303 (71.15)  454 (14.03) | 439 (16.72)  1882 (71.67)  305 (11.61) | 74 (9.40)  454 (57.69)  259 (32.91) | 1608 (14.04)  8099 (70.74)  1742 (15.22) | 298 / 11747 (2.54) |
| **Religiousness**  No/Passive  Active | 4079 (90.42)  432 (9.58) | 2493 (83.38)  497 (16.62) | 2066 (82.02)  453 (17.98) | 650 (88.56)  84 (11.44) | 9288 (86.37)  1466 (13.63) | 993 / 11747 (8.45) |
| **Loss of loved ones**  No  Yes | 4154 (84.95)  736 (15.05) | 2760 (82.44)  588 (17.56) | 2137 (79.35)  556 (20.65) | 700 (85.78)  116 (14.22) | 9751 (83.01)  1996 (16.99) | 0 / 11747 (0.00) |
| **Loneliness**  Not lonely  Lonely | 4341 (91.08)  425 (8.92) | 2906 (89.83)  329 (10.17) | 2247 (85.76)  373 (14.24) | 709 (89.52)  83 (10.48) | 10203 (89.40)  1210 (10.60) | 334 / 11747 (2.84) |
| **Sleep Quality**  Bad quality  Good quality | 1460 (30.49)  3328 (69.51) | 1085 (33.31)  2172 (66.69) | 888 (34.10)  1716 (65.90) | 234 (30.08)  544 (69.92) | 3667 (32.09)  7760 (67.91) | 320 / 11747 (2.72) |
| **Depression (GDS ≥2)**  No  Yes | 4309 (88.90)  538 (11.10) | 3078 (92.71)  242 (7.29) | 2478 (93.09)  184 (6.91) | 719 (89.76)  82 (10.24) | 10584 (91.01)  1046 (8.99) | 117 / 11747 (1.00) |
| **CVD**  No  Yes | 1467 (30.84)  3290 (69.16) | 1173 (36.24)  2064 (63.76) | 944 (36.15)  1667 (63.85) | 303 (38.45)  485 (61.55) | 3887 (34.12)  7506 (65.88) | 354 / 11747 (3.01) |
| **Polypharmacy**  No  Yes | 2813 (68.66)  1284 (31.34) | 2154 (71.92)  841 (28.08) | 1535 (64.88)  831 (35.12) | 573 (75.39)  187 (24.61) | 7075 (69.24)  3143 (30.76) | 1529 / 11747 (13.02) |
| **ADL independence**  No  Yes | 1870 (39.99)  2806 (60.01) | 1445 (45.41)  1737 (54.59) | 1097 (42.54)  1482 (57.46) | 327 (41.71)  457 (58.29) | 4739 (42.23)  6482 (57.77) | 526 / 11747 (4.48) |
| **Frailty**  Not frail  Frail | 2636 (56.51)  2029 (43.49) | 2095 (65.37)  1110 (34.63) | 1544 (59.45)  1053 (40.55) | 518 (65.65)  271 (34.35) | 6793 (60.35)  4463 (39.65) | 491 / 11747 (4.18) |
| **Physical activity**  <150 min/week  ≥150 min/week | 1806 (38.21)  2920 (61.79) | 1046 (33.03)  2121 (66.97) | 930 (36.54)  1615 (63.46) | 264 (35.92)  471 (64.08) | 4046 (36.21)  7127 (63.79) | 574 / 11747 (4.89) |
| **BMI**  <23 kg/m^2^  23-29.9 kg/m^2^  ≥30 kg/m^2^ | 991 (21.25)  2841 (60.93)  831 (17.82) | 592 (18.79)  1933 (61.35)  626 (19.87) | 400 (15.37)  1591 (61.12)  612 (23.51) | 132 (16.79)  505 (64.25)  149 (18.96) | 2115 (18.88)  6870 (61.32)  2218 (19.80) | 544 / 11747 (4.63) |
| **Subjective health**  Fair/bad  Good  Very good/excellent | 1499 (31.12)  1606 (33.34)  1712 (35.54) | 1039 (31.32)  1116 (33.64)  1162 (35.03) | 944 (35.70)  573 (21.67)  1127 (42.62) | 245 (30.86)  325 (40.93)  224 (28.21) | 3727 (32.21)  3620 (31.28)  4225 (36.51) | 175 / 11747 (1.49) |
|  | m (sd) | m (sd) | m (sd) | m (sd) | m (sd) |  |
| **Inner strength scale** | 80.23 (12.53) | 78.60 (12.44) | 77.91 (13.03) | 79.06 (12.88) | 79.15 (12.68) | 1711 / 11747 (14.57) |

# **Supplementary Table S2**. Descriptive statistics for all covariates – imputed data (imputations = 20)**.**

|  | **Westrobothnia**  **n (%)** | **Ostrobothnia**  **n (%)** | **South Ostrobothnia**  **n (%)** | **Åland**  **n (%)** | **Total**  **n (%)** |
| --- | --- | --- | --- | --- | --- |
| **Total** | 97800 (41.63) | 66960 (28.50) | 53860 (22.93) | 16320 (6.95) | 234940 (100.00) |
| **Gender**  Men  Women | 48420 (49.51)  49380 (50.49) | 29780 (44.47)  37180 (55.53) | 23200 (43.07)  30660 (56.93) | 7700 (47.18)  8620 (52.82) | 109100 (46.44)  125840 (53.56) |
| **Age group**  65  70  75  80  85  90 | 39180 (40.06)  19240 (19.67)  20500 (20.96)  10000 (10.22)  6580 (6.73)  2300 (2.35) | 15340 (22.91)  17680 (26.40)  17680 (26.40)  8020 (11.98)  5620 (8.39)  2620 (3.91) | 14720 (27.33)  14460 (26.85)  11720 (21.76)  6280 (11.66)  4460 (8.28)  2220 (4.12) | 4640 (28.43)  4060 (24.88)  4160 (25.49)  2240 (13.73)  1000 (6.13)  220 (1.35) | 73880 (31.45)  55440 (23.60)  54060 (23.01)  26540 (11.30)  17660 (7.52)  7360 (3.13) |
| **Marital status**  Single/divorced/widow  Married/partner | 29658 (30.33)  68142 (69.67) | 18287 (27.31)  48673 (72.69) | 16871 (31.32)  36989 (68.68) | 4861 (29.79)  11459 (70.21) | 69677 (29.66)  165263 (70.34) |
| **Education level**  Primary/ lower 2ndary  Upper secondary  Higher education | 32990 (33.73)  36090 (36.90)  28720 (29.37) | 26249 (39.20)  32637 (48.74)  8074 (12.06) | 18492 (34.33)  29939 (55.59)  5429 (10.08) | 3700 (22.67)  9225 (56.53)  3395 (20.80) | 81431 (34.66)  107891 (45.92)  45618 (19.42) |
| **Income after tax**  ≤1000 €  1001-2000 €  ˃2000 € | 12692 (12.98)  70436 (72.02)  14672 (15.00) | 10133 (15.13)  47554 (71.02)  9273 (13.85) | 9057 (16.82)  38585 (71.64)  6218 (11.54) | 1574 (9.64)  9484 (58.11)  5262 (32.24) | 33456 (14.24)  166059 (70.68)  35425 (15.08) |
| **Religiousness**  No/Passive  Active | 88252 (90.24)  9548 (9.76) | 55817 (83.36)  11143 (16.64) | 44091 (81.86)  9769 (18.14) | 14358 (87.98)  1962 (12.02) | 202518 (86.20)  32422 (13.80) |
| **Loss of loved ones**  No  Yes | 83080 (84.95)  14720 (15.05) | 55200 (82.44)  11760 (17.56) | 42740 (79.35)  11120 (20.65) | 14000 (85.78)  2320 (14.22) | 195020 (83.01)  39920 (16.99) |
| **Loneliness**  Not lonely  Lonely | 88894 (90.89)  8906 (9.11) | 59910 (89.47)  7050 (10.53) | 46013 (85.43)  7847 (14.57) | 14507 (88.89)  1813 (11.11) | 209324 (89.10)  25616 (10.90) |
| **Sleep Quality**  Bad quality  Good quality | 29903 (30.58)  67897 (69.42) | 22342 (33.37)  44618 (66.63) | 18453 (34.26)  35407 (65.74) | 4968 (30.44)  11352 (69.56) | 75666 (32.21)  159274 (67.79) |
| **Depression (GDS ≥2)**  No  Yes | 86628 (88.58)  11172 (11.42) | 61655 (92.08)  5305 (7.92) | 49593 (92.08)  4267 (7.92) | 14590 (89.40)  1730 (10.60) | 212466 (90.43)  22474 (9.57) |
| **CVD**  No  Yes | 31188 (31.89)  66612 (68.11) | 24301 (36.29)  42659 (63.71) | 19724 (36.62)  34136 (63.38) | 6317 (38.71)  10003 (61.29) | 81530 (34.70)  153410 (65.30) |
| **Polypharmacy**  No  Yes | 67045 (68.55)  30755 (31.45) | 48504 (72.44)  18456 (27.56) | 36169 (67.15)  17691 (32.85) | 12284 (75.27)  4036 (24.73) | 164002 (69.81)  70938 (30.19) |
| **ADL independence**  No  Yes | 38463 (39.33)  59337 (60.67) | 30195 (45.09)  36765 (54.91) | 22698 (42.14)  31162 (57.86) | 6760 (41.42)  9560 (58.58) | 98116 (41.76)  136824 (58.24) |
| **Frailty**  Not frail  Frail | 56449 (57.72)  41351 (42.28) | 43954 (65.64)  23006 (34.36) | 32373 (60.11)  21487 (39.89) | 10720 (65.69)  5600 (34.31) | 143496 (61.08)  91444 (38.92) |
| **Physical activity**  <150 min/week  ≥150 min/week | 37659 (38.51)  60141 (61.49) | 22686 (33.88)  44274 (66.12) | 19865 (36.88)  33995 (63.12) | 5799 (35.53)  10521 (64.47) | 86009 (36.61)  148931 (63.39) |
| **BMI**  <23 kg/m^2^  23-29.9 kg/m^2^  ≥30 kg/m^2^ | 20813 (21.28)  59273 (60.61)  17714 (18.11) | 12635 (18.87)  40878 (61.05)  13447 (20.08) | 8352 (15.51)  32755 (60.82)  12753 (23.68) | 2756 (16.89)  10440 (63.97)  3124 (19.14) | 44556 (18.96)  143346 (61.01)  47038 (20.02) |
| **Subjective health**  Fair/bad  Good  Very good/excellent | 30628 (31.32)  32528 (33.26)  34644 (35.42) | 21048 (31.43)  22490 (33.59)  23422 (34.98) | 19341 (35.91)  11716 (21.75)  22803 (42.34) | 5087 (31.17)  6637 (40.67)  4596 (28.16) | 76104 (32.39)  73371 (31.23)  85465 (36.38) |
|  | m (sd) | m (sd) | m (sd) | m (sd) | m (sd) |
| **Inner strength scale** | 79.59 (13.41) | 77.54 (13.75) | 77.09 (14.07) | 78.30 (13.74) | 78.35 (13.73) |

# **Supplementary Table S3.** RRRs, 95% confidence intervals (CIs), and p-values from standard multinomial regression models comparing current abstinence and hazardous drinking to low-risk drinking among men.

| **Covariates** | **Model 1 (Bivariate)** | | **Model 2 (Sociodemographic)** | | | **Model 3 sociodemographic +**  **other psychosocial functioning** | | | **Model 4 sociodemographic +**  **other psychosocial variables +**  **Physical health/physical functioning** | | |
| --- | --- | --- | --- | --- | --- | --- | --- | --- | --- | --- | --- |
|  | **RRR (95%CI)** | **p value** | **RRR** | **95%CI** | **p value** | **RRR** | **95%CI** | **p value** | **RRR** | **95%CI** | **p value** |
| **Current abstinence** |  |  |  |  |  |  |  |  |  |  |  |
| **Age group**  65  70  75  80  85  90 | 1/Ref  1.16 (0.94-1.42)  **1.26 (1.04-1.52)**  **1.93 (1.54-2.42)**  **2.43 (1.88-3.15)**  **4.02 (2.68-6.0**4) | 0.167  **0.018**  **<0.001**  **<0.001**  **<0.001** | 1  1.10  1.13  **1.68**  **2.03**  **3.01** | 0.89-1.35  0.93-1.38  **1.33-2.12**  **1.56-2.66**  **1.98-4.59** | 0.377  0.212  **<0.001**  **<0.001**  **<0.001** | 1  1.07  1.13  **1.59**  **1.89**  **2.84** | 0.87-1.33  0.93-1.38  **1.25-2.02**  **1.43-2.48**  **1.84-4.37** | 0.504  0.231  **<0.001**  **<0.001**  **<0.001** | 1  1.04  1.00  **1.29**  **1.36**  **1.82** | 0.84-1.29  0.82-1.23  **1.01-1.65**  **1.02-1.81**  **1.16-2.85** | 0.703  0.982  **0.043**  **0.039**  **0.009** |
| **Marital status**  Single/divorced/widow  Married/partner | 1  **0.68 (0.58-0.81)** | **<0.001** | 1  **0.78** | **0.65-0.93** | **0.005** | 1  **0.78** | **0.65-0.93** | **0.007** | 1  **0.74** | **0.61-0.89** | **0.001** |
| **Education level**  Primary/ lower 2ndary  Upper secondary  Higher education | 1  **0.63 (0.54-0.73)**  **0.50 (0.40-0.61)** | **<0.001**  **<0.001** | 1  **0.76**  **0.76** | **0.65-0.89**  **0.61-0.96** | **0.001**  **0.024** | 1  **0.77**  **0.71** | **0.65-0.90**  **0.56-0.90** | **0.001**  **0.004** | 1  **0.80**  **0.76** | **0.68-0.93**  **0.59-0.97** | **0.005**  **0.025** |
| **Income after tax**  ≤1000 €  1001-2000 €  ˃2000 € | 1  **0.63 (0.50-0.81)**  **0.28 (0.21-0.38)** | **<0.001**  **<0.001** | 1  **0.74**  **0.39** | **0.57-0.95**  **0.29-0.54** | **0.016**  **<0.001** | 1  **0.76**  **0.42** | **0.59-0.98**  **0.30-0.58** | **0.034**  **<0.001** | 1  0.81  **0.47** | 0.62-1.05  **0.34-0.65** | 0.107  **<0.001** |
| **Residence region**  Westrobothnia  Ostrobothnia  South Ostrobothnia  Åland | 1  1.17 (0.99-1.38)  **1.42 (1.20-1.69)**  **0.66 (0.48-0.92)** | 0.064  **<0.001**  **0.015** | 1  1.11  **1.36**  0.79 | 0.93-1.32  **1.13-1.64**  0.56-1.11 | 0.248  **0.001**  0.168 | 1  1.02  **1.26**  0.71 | 0.85-1.22  **1.05-1.52**  0.51-1.01 | 0.845  **0.015**  0.057 | 1  1.07  **1.30**  0.74 | 0.89-1.29  **1.08-1.58**  0.52-1.05 | 0.449  **0.007**  0.090 |
| **Religiousness**  No/Passive  Active | 1  **2.62 (2.15-3.18)** | **<0.001** |  |  |  | 1  **2.83** | **2.31-3.48** | **<0.001** | 1  **2.88** | **2.34-3.55** | **<0.001** |
| **Loss of loved ones**  No  Yes | 1  1.10 (0.90-1.34) | 0.361 |  |  |  | 1  0.87 | 0.71-1.08 | 0.214 | 1  0.88 | 0.71-1.09 | 0.237 |
| **Loneliness**  Not lonely  Lonely | 1  **1.54 (1.22-1.94)** | **<0.001** |  |  |  | 1  1.02 | 0.78-1.33 | 0.878 | 1  0.97 | 0.74-1.28 | 0.846 |
| **Inner strength scale** | **0.99 (0.98-0.99)** | **<0.001** |  |  |  | **0.99** | **0.99-1.00** | **0.021** | 0.99 | 0.99-1.00 | 0.282 |
| **Sleep quality**  Bad quality  Good quality | 1  **0.81 (0.70-0.95)** | **0.008** |  |  |  | 1  0.90 | 0.77-1.07 | 0.240 | 1  0.98 | 0.83-1.17 | 0.845 |
| **Depression (GDS ≥2)**  No  Yes | 1  **1.87 (1.47-2.38)** | **<0.001** |  |  |  | 1  **1.52** | **1.15-2.01** | **0.003** | 1  1.28 | 0.96-1.71 | 0.090 |
| **CVD**  No  Yes | 1  **1.23 (1.06-1.43)** | **0.007** |  |  |  |  |  |  | 1  0.94 | 0.79-1.12 | 0.472 |
| **Polypharmacy**  No  Yes | 1  **1.82 (1.57-2.10)** | **<0.001** |  |  |  |  |  |  | 1  **1.38** | **1.16-1.64** | **<0.001** |
| **ADL independence**  No  Yes | 1  **0.52 (0.45-0.60)** | **<0.001** |  |  |  |  |  |  | 1  **0.68** | **0.58-0.80** | **<0.001** |
| **Frailty**  Not frail  Frail | 1  **2.05 (1.77-2.38)** | **<0.001** |  |  |  |  |  |  | 1  **1.28** | **1.05-1.56** | **0.013** |
| **Physical activity**  <150 min/week  ≥150 min/week | 1  **0.60 (0.52-0.69)** | **<0.001** |  |  |  |  |  |  | 1  0.86 | 0.73-1.02 | 0.090 |
| **BMI**  <23 kg/m^2^  23-29.9 kg/m^2^  ≥30 kg/m^2^ | 1  0.90 (0.74-1.08)  0.92 (0.72-1.16) | 0.258  0.478 |  |  |  |  |  |  | 1  0.93  0.78 | 0.76-1.14  0.60-1.01 | 0.492  0.064 |
| **Subjective health**  Fair/bad  Good  Very good/excellent | 1  **0.60 (0.51-0.71)**  **0.54 (0.46-0.64)** | **<0.001**  **<0.001** |  |  |  |  |  |  | 1  0.92  0.98 | 0.75-1.12  0.79-1.22 | 0.421  0.879 |
| **Hazardous drinking** |  |  |  |  |  |  |  |  |  |  |  |
| **Age group**  65  70  75  80  85  90 | 1/Ref  0.88 (0.75-1.03)  **0.52 (0.44-0.62)**  **0.42 (0.33-0.53)**  **0.30 (0.21-0.43)**  **0.29 (0.15-0.55)** | 0.122  **<0.001**  **<0.001**  **<0.001**  **<0.001** | 1  0.93  **0.55**  **0.44**  **0.32**  **0.30** | 0.79-1.10  **0.46-0.66**  **0.34-0.56**  **0.22-0.45**  **0.16-0.58** | 0.379  **<0.001**  **<0.001**  **<0.001**  **<0.001** | 1  0.94  **0.55**  **0.44**  **0.32**  **0.30** | 0.80-1.11  **0.46-0.66**  **0.34-0.56**  **0.22-0.45**  **0.16-0.58** | 0.466  **<0.001**  **<0.001**  **<0.001**  **<0.001** | 1  0.96  **0.56**  **0.46**  **0.33**  **0.33** | 0.81-1.13  **0.47-0.67**  **0.35-0.59**  **0.23-0.48**  **0.17-0.65** | 0.614  **<0.001**  **<0.001**  **<0.001**  **<0.001** |
| **Marital status**  Single/divorced/widow  Married/partner | 1  0.86 (0.74-1.01) | 0.074 | 1  **0.80** | **0.68-0.94** | **0.009** | 1  **0.82** | **0.69-0.97** | **0.020** | 1  0.84 | 0.71-1.00 | 0.051 |
| **Education level**  Primary/ lower 2ndary  Upper secondary  Higher education | 1  **1.34 (1.15-1.55)**  **1.51 (1.06-1.82)** | **<0.001**  **<0.001** | 1  1.11  1.14 | 0.95-1.30  0.93-1.40 | 0.176  0.202 | 1  1.12  1.18 | 0.96-1.31  0.96-1.45 | 0.145  0.109 | 1  1.11  1.18 | 0.95-1.30  0.96-1.45 | 0.195  0.116 |
| **Income after tax**  ≤1000 €  1001-2000 €  ˃2000 € | 1  1.10 (0.84-1.45)  **1.55 (1.16-2.08)** | 0.481  **0.003** | 1  1.00  1.27 | 0.76-1.33  0.93-1.72 | 0.987  0.127 | 1  1.03  1.35 | 0.78-1.37  0.99-1.85 | 0.826  0.055 | 1  1.02  1.34 | 0.76-1.35  0.98-1.84 | 0.908  0.066 |
| **Residence region**  Westrobothnia  Ostrobothnia  South Ostrobothnia  Åland | 1  **0.76 (0.65-0.89)**  **0.83 (0.70-0.98)**  1.12 (0.88-1.43) | **0.001**  **0.027**  0.348 | 1  **0.84**  0.87  1.17 | **0.72-0.99**  0.73-1.03  0.91-1.50 | **0.038**  0.106  0.231 | 1  **0.85**  0.88  1.16 | **0.72-1.00**  0.73-1.05  0.90-1.49 | **0.047**  0.143  0.254 | 1  **0.84**  0.87  1.13 | **0.71-1.00**  0.73-1.05  0.88-1.46 | **0.042**  0.149  0.349 |
| **Religiousness**  No/Passive  Active | 1  **0.43 (0.32-0.57)** | **<0.001** |  |  |  | 1  **0.45** | **0.33-0.60** | **<0.001** | 1  **0.44** | **0.33-0.59** | **<0.001** |
| **Loss of loved ones**  No  Yes | 1  1.01 (0.84-1.21) | 0.947 |  |  |  | 1  1.07 | 0.88-1.29 | 0.518 | 1  1.07 | 0.88-1.29 | 0.517 |
| **Loneliness**  Not lonely  Lonely | 1  0.86 (0.67-1.10) | 0.220 |  |  |  | 1  0.79 | 0.60-1.03 | 0.084 | 1  0.77 | 0.59-1.02 | 0.066 |
| **Inner strength scale** | 1.00 (0.99-1.00) | 0.337 |  |  |  | 1.00 | 0.99-1.00 | 0.064 | 0.99 | 0.99-1.00 | 0.055 |
| **Sleep quality**  Bad quality  Good quality | 1  **0.80 (0.70-0.93)** | **0.003** |  |  |  | 1  **0.79** | **0.68-0.92** | **0.003** | 1  **0.79** | **0.68-0.92** | **0.003** |
| **Depression (GDS ≥2)**  No  Yes | 1  1.22 (0.96-155) | 0.106 |  |  |  | 1  1.17 | 0.89-1.52 | 0.260 | 1  1.20 | 0.91-1.58 | 0.193 |
| **CVD**  No  Yes | 1  1.10 (0.96-1.26) | 0.179 |  |  |  |  |  |  | 1  **1.19** | **1.02-1.39** | **0.025** |
| **Polypharmacy**  No  Yes | 1  **0.80 (0.69-0.94)** | **0.006** |  |  |  |  |  |  | 1  **0.83** | **0.69-1.00** | **0.045** |
| **ADL independence**  No  Yes | 1  **1.35 (1.19-1.53)** | **<0.001** |  |  |  |  |  |  | 1  **1.15** | **1.00-1.33** | **0.049** |
| **Frailty**  Not frail  Frail | 1  0.96 (0.83-1.11) | 0.614 |  |  |  |  |  |  | 1  1.20 | 1.00-1.44 | 0.056 |
| **Physical activity**  <150 min/week  ≥150 min/week | 1  1.00 (0.87-1.16) | 0.960 |  |  |  |  |  |  | 1  0.96 | 0.81-1.12 | 0.579 |
| **BMI**  <23 kg/m^2^  23-29.9 kg/m^2^  ≥30 kg/m^2^ | 1  **1.38 (1.14-1.67)**  **1.73 (1.38-2.16)** | **0.001**  **<0.001** |  |  |  |  |  |  | 1  **1.37**  **1.66** | **1.13-1.67**  **1.31-2.11** | **0.002**  **<0.001** |
| **Subjective health**  Fair/bad  Good  Very good/excellent | 1  **1.25 (1.06-1.48)**  1.15 (0.98-1.35) | **0.008**  0.092 |  |  |  |  |  |  | 1  **1.26**  1.15 | **1.04-1.52**  0.94-1.42 | **0.017**  0.169 |

# **Supplementary Table S4.** RRRs. 95% confidence intervals (CIs), and p-values from mixed-effects multinomial regression models comparing current abstinence and hazardous drinking to low-risk drinking among women.

| **Covariates** | **Model 1 (Bivariate)** | | **Model 2 (Sociodemographic)** | | | **Model 3 sociodemographic +**  **other psychosocial functioning** | | | **Model 4 sociodemographic + other psychosocial variables + Physical health/physical functioning** | | |
| --- | --- | --- | --- | --- | --- | --- | --- | --- | --- | --- | --- |
|  | **RRR (95%CI)** | **p-value** | **RRR** | **95%CI** | **p value** | **RRR** | **95%CI** | **p value** | **RR** | **95%CI** | **p value** |
| **Current abstinence** |  |  |  |  |  |  |  |  |  |  |  |
| **Age group**  65  70  75  80  85  90 | 1  **1.38 (1.18-1.61)**  **2.19 (1.87-2.56)**  **3.13 (2.59-3.79)**  **4.44 (3.54-5.55)**  **8.76 (6.21-12.36)** | **<0.001**  **<0.001**  **<0.001**  **<0.001**  **<0.001** | 1  **1.31**  **1.88**  **2.48**  **3.30**  **5.76** | **1.12-1.57**  **1.60-2.20**  **2.04-3.02**  **2.62-4.17**  **4.03-8.22** | **0.001**  **<0.001**  **<0.001**  **<0.001**  **<0.001** | 1  **1.29**  **1.77**  **2.26**  **3.00**  **5.20** | **1.10-1.52**  **1.50-2.09**  **1.85-2.77**  **2.37-3.81**  **3.61-7.49** | **0.002**  **<0.001**  **<0.001**  **<0.001**  **<0.001** | 1  **1.26**  **1.57**  **1.71**  **2.00**  **2.91** | **1.07-1.49**  **1.33-1.86**  **1.38-2.11**  **1.55-2.58**  **1.98-4.27** | **0.006**  **<0.001**  **<0.001**  **<0.001**  **<0.001** |
| **Marital status**  Single/divorced/widow  Married/partner | 1  **0.55 (0.50-0.62)** | **<0.001** | 1  **0.71** | **0.63-0.81** | **<0.001** | 1  **0.71** | **0.63-0.81** | **<0.001** | 1  **0.72** | **0.63-0.82** | **<0.001** |
| **Education level**  Primary/ lower 2ndary  Upper secondary  Higher education | 1  **0.59 (0.52-0.67)**  **0.35 (0.29-0.41)** | **<0.001**  **<0.001** | 1  **0.83**  **0.59** | **0.72-0.94**  **0.49-0.70** | **0.005**  **<0.001** | 1  **0.78**  **0.52** | **0.68-0.90**  **0.43-0.63** | **<0.001**  **<0.001** | 1  **0.81**  **0.56** | **0.71-0.93**  **0.46-0.68** | **0.004**  **<0.001** |
| **Income**  ≤1000 €  1001-2000 €  ˃2000 € | 1  **0.48 (0.42-0.56)**  **0.24 (0.18-0.31)** | **<0.001**  **<0.001** | 1  **0.55**  **0.36** | **0.47-0.64**  **0.27-0.48** | **<0.001**  **<0.001** | 1  **0.57**  **0.39** | **0.49-0.66**  **0.29-0.53** | **<0.001**  **<0.001** | 1  **0.61**  **0.45** | **0.52-0.71**  **0.33-0.61** | **<0.001**  **<0.001** |
| **Religiousness**  No/Passive  Active | 1  **2.93 (2.53-3.41)** | **<0.001** |  |  |  | 1  **3.17** | **2.70-3.72** | **0.000** | 1  **3.27** | **2.78-3.85** | **<0.001** |
| **Loss of loved ones**  No  Yes | 1  1.00 (0.87-1.14) | 0.997 |  |  |  | 1  1.16 | 0.93-1.44 | 0.193 | 1  **0.83** | **0.72-0.97** | **0.019** |
| **Loneliness**  Not lonely  Lonely | 1  **1.67 (1.42-1.97)** | **<0.001** |  |  |  | 1  0.85 | 0.69-1.04 | 0.107 | 1  1.12 | 0.92-1.37 | 0.244 |
| **Inner strength scale** | **0.99 (0.98-0.99)** | **<0.001** |  |  |  | **0.99** | **0.99-1.00** | **0.003** | 0.99 | 0.99-1.00 | 0.565 |
| **Sleep quality**  Bad quality  Good quality | 1  0.97 (0.87-1.09) | 0.639 |  |  |  | 1  **0.86** | **0.76-0.97** | **0.012** | 1  **1.19** | **1.05-1.36** | **0.008** |
| **Depression (GDS ≥ 2)**  No  Yes | 1  **1.61 (1.35-1.92)** | **<0.001** |  |  |  | 1  **1.29** | **1.05-1.58** | **0.015** | 1  1.10 | 0.88-1.36 | 0.408 |
| **CVD**  No  Yes | 1  **1.58 (1.41-1.77)** | **<0.001** |  |  |  |  |  |  | 1  1.03 | 0.90-1.18 | 0.681 |
| **Polypharmacy**  No  Yes | 1  **2.22 (1.96-2.51)** | **<0.001** |  |  |  |  |  |  | 1  **1.37** | **1.17-1.61** | **<0.001** |
| **ADL independence**  No  Yes | 1  **0.37 (0.33-0.42)** | **<0.001** |  |  |  |  |  |  | 1  **0.63** | **0.55-0.72** | **<0.001** |
| **Frailty**  Not frail  Frail | 1  **2.15 (1.92-2.41)** | **<0.001** |  |  |  |  |  |  | 1  1.05 | 0.90-1.23 | 0.522 |
| **Physical activity**  <150 min/week  ≥150 min/week | 1  **0.53 (0.48-0.60)** | **<0.001** |  |  |  |  |  |  | 1  0.92 | 0.80-1.05 | 0.216 |
| **BMI**  <23 kg/m^2^  23-29.9 kg/m^2^  ≥30 kg/m^2^ | 1  0.90 (0.78-1.04)  1.00 (0.84-1.18) | 0.149  0.975 |  |  |  |  |  |  | 1  **0.82**  **0.75** | **0.70-0.96**  **0.62-0.91** | **0.013**  **0.004** |
| **Subjective health**  Fair/bad  Good  Very good/excellent | 1  **0.46 (0.40-0.53)**  **0.37 (0.32-.42)** | **<0.001**  **<0.001** |  |  |  |  |  |  | 1  **0.70**  **0.70** | **0.59-0.83**  **0.58-0.84** | **0.000**  **0.000** |
| **Hazardous drinking** |  |  |  |  |  |  |  |  |  |  |  |
| **Age group**  65  70  75  80  85  90 | 1  0.82 (0.66-1.01)  **0.66 (0.52-0.84)**  **0.41 (0.28-0.62)**  **0.27 (0.14-0.50)**  0.57 (0.25-1.27) | 0.061  **<0.001**  **<0.001**  **<0.001**  0.171 | 1  0.85  **0.70**  **0.45**  **0.30**  0.66 | 0.69-1.05  **0.55-0.90**  **0.30-0.68**  **0.16-0.56**  0.29-1.49 | 0.138  **0.006**  **<0.001**  **<0.001**  0.317 | 1  0.86  **0.70**  **0.44**  **0.28**  0.63 | 0.70-1.07  **0.55-0.90**  **0.29-0.66**  **0.15-0.54**  0.28-1.44 | 0.178  **0.006**  **<0.001**  **<0.001**  0.276 | 1  0.86  **0.69**  **0.41**  **0.26**  0.55 | 0.69-1.06  **0.53-0.88**  **0.27-0.62**  **0.14-0.50**  0.24-1.27 | 0.159  **0.004**  **<0.001**  **<0.001**  0.163 |
| **Marital status**  Single/divorced/widow  Married/partner | 1  1.18 (0.96-1.41) | 0.112 | 1  1.01 | 0.83-1.23 | 0.936 | 1  1.07 | 0.88-1.32 | 0.495 | 1  1.07 | 0.87-1.31 | 0.532 |
| **Education level**  Primary/ lower 2ndary  Upper secondary  Higher education | 1  1.14 (0.91-1.44)  **1.93 (1.52-2.46)** | 0.256  **<0.001** | 1  1.02  **1.49** | 0.80-1.29  **1.15-1.94** | 0.885  **0.003** | 1  1.05  **1.57** | 0.83-1.34  **1.21-2.05** | 0.657  **0.001** | 1  1.06  **1.56** | 0.84-1.34  **1.19-2.03** | 0.631  **0.001** |
| **Income**  ≤1000 €  1001-2000 €  ˃2000 € | 1  0.96 (0.74-1.24)  **2.08 (1.50-2.88)** | 0.767  **<0.001** | 1  0.85  **1.48** | 0.65-1.11  **1.04-2.10** | 0.224  **0.030** | 1  0.88  **1.61** | 0.68-1.15  **1.13-2.30** | 0.357  **0.009** | 1  0.88  **1.58** | 0.67-1.15  **1.10-2.27** | 0.356  **1.013** |
| **Religiousness**  No/Passive  Active | 1  **0.69 (0.50-0.96)** | **0.028** |  |  |  | 1  0.72 | 0.52-1.01 | 0.054 | 1  0.73 | 0.52-1.02 | 0.062 |
| **Loss of loved ones**  No  Yes | 1  1.06 (0.86-1.31) | 0.585 |  |  |  | 1  1.16 | 0.93-1.44 | 0.193 | 1  1.16 | 0.93-1.44 | 0.194 |
| **Loneliness**  Not lonely  Lonely | 1  1.05 (0.79-1.39) | 0.724 |  |  |  | 1  0.85 | 0.69-1.04 | 0.107 | 1  0.99 | 0.72-1.35 | 0.950 |
| **Inner strength scale** | **0.99 (0.99-1.00)** | **0.039** |  |  |  | **0.99** | **0.99-1.00** | **0.003** | **0.99** | **0.98-1.00** | **0.002** |
| **Sleep quality**  Bad quality  Good quality | 1  0.93 (0.78-1.12) | 0.434 |  |  |  | 1  **0.86** | **0.76-0.97** | **0.012** | 1  0.95 | 0.78-1.15 | 0.583 |
| **Depression (GDS ≥ 2)**  No  Yes | 1  **1.46 (1.11-1.94)** | **0.008** |  |  |  | 1  **1.29** | **1.05-1.58** | **0.015** | 1  **1.43** | **1.04-1.97** | **0.030** |
| **CVD**  No  Yes | 1  1.00 (0.84-1.19) | 0.984 |  |  |  |  |  |  | 1  **1.22** | **1.00-1.48** | **0.047** |
| **Polypharmacy**  No  Yes | 1  **0.72 (0.57-0.91)** | **0.007** |  |  |  |  |  |  | 1  **0.76** | **0.58-0.99** | **0.040** |
| **ADL independence**  No  Yes | 1  1.11 (0.91-1.36) | 0.314 |  |  |  |  |  |  | 1  0.92 | 0.73-1.15 | 0.466 |
| **Frailty**  Not frail  Frail | 1  1.02 (0.85-1.22) | 0.847 |  |  |  |  |  |  | 1  1.17 | 0.93-1.47 | 0.170 |
| **Physical activity**  <150 min/week  ≥150 min/week | 1  1.00 (0.83-1.20) | 0.983 |  |  |  |  |  |  | 1  0.88 | 0.71-1.08 | 0.225 |
| **BMI**  <23 kg/m^2^  23-29.9 kg/m^2^  ≥30 kg/m^2^ | 1  1.04 (0.83-1.30)  0.81 (0.60-1.08) | 0.745  0.152 |  |  |  |  |  |  | 1  1.07  0.82 | 0.85-1.35  0.60-1.12 | 0.560  0.210 |
| **Subjective health**  Fair/bad  Good  Very good/excellent | 1  1.10 (0.87-1.40)  **1.29 (1.03-1.62)** | 0.431  **0.025** |  |  |  |  |  |  | 1  1.09  1.30 | 0.83-1.43  0.97-1.74 | 0.537  0.083 |
| **Var [residence region] (SE)** | M1: empty model  **0.051 (0.04)** | | M2: Sociodemographic **0.037 (0.03)** | | | M3: sociodemographic + other psychosocial functioning  **0.031 (0.03)** | | | M4: sociodemographic + other psychosocial variables + Physical health/physical functioning  **0.033 (0.03)** | | |

# **Supplementary Table S5.** ORs, 95% confidence intervals (CIs), and p-values from ordinary logistic regression models for heavy episodic drinking among men.

| **Covariates** | **Model 1 (Bivariate)** | | **Model 2 (Sociodemographic)** | | | **Model 3 sociodemographic +**  **other psychosocial functioning** | | | **Model 4 sociodemographic +**  **other psychosocial variables +**  **Physical health/physical functioning** | | |
| --- | --- | --- | --- | --- | --- | --- | --- | --- | --- | --- | --- |
|  | **OR (95%CI)** | **p value** | **OR** | **95%CI** | **p value** | **OR** | **95%CI** | **p value** | **OR** | **95%CI** | **p value** |
| **Age group**  65  70  75  80  85  90 | 1  **0.79 (0.65-0.96)**  **0.47 (0.38-0.59)**  **0.33 (0.24-0.47)**  **0.27 (0.17-0.42)**  **0.14 (0.05-0.39)** | **0.019**  **<0.001**  **<0.001**  **<0.001**  **<0.001** | 1  0.84  **0.50**  **0.35**  **0.27**  **0.14** | 0.69-1.03  **0.40- 0.63**  **0.25- 0.50**  **0.17-0.43**  **0.05-0.39** | 0.093  **<0.001**  **<0.001**  **<0.001**  **<0.001** | 1  0.86  **0.51**  **0.36**  **0.28**  **0.14** | 0.70-1.05  **0.40-0.64**  **0.25-0.51**  **0.17-0.44**  **0.05-0.39** | 0.129  **<0.001**  **<0.001**  **<0.001**  **<0.001** | 1  0.87  **0.52**  **0.37**  **0.29**  **0.16** | 0.71-1.07  **0.41-0.66**  **0.26-0.53**  **0.18-0.47**  **0.06-0.44** | 0.177  **<0.001**  **<0.001**  **<0.001**  **<0.001** |
| **Marital status**  Single/divorced/widow  Married/partner | 1  **0.78 (0.64-0.94)** | **0.010** | 1  **0.70** | **0.58-086** | **<0.001** | 1  **0.75** | **0.61-0.93** | **0.007** | 1  **0.79** | **0.64-0.97** | **0.027** |
| **Education level**  Primary/ lower 2ndary  Upper secondary  Higher education | 1  **1.40 (1.17-1.69)**  **1.27 (1.00-1.62)** | **<0.001**  **0.047** | 1  1.12  0.88 | 0.92-136  0.67-1.14 | 0.256  0.330 | 1  1.12  0.91 | 0.92-1.37  0.69-1.18 | 0.242  0.469 | 1  1.10  0.90 | 0.90-1.34  0.68-1.18 | 0.350  0.440 |
| **Income**  ≤1000 €  1001-2000 €  ˃2000 € | 1  0.89 (0.65-1.22)  1.28 (0.92-1.79) | 0.473  0.141 | 1  0.77  1.04 | 0.56-1.06  0.73-1.48 | 0.108  0.816 | 1  0.80  1.14 | 0.58-1.11  0.79-1.63 | 0.188  0.479 | 1  0.79  1.13 | 0.57-1.10  0.78-1.63 | 0.164  0.509 |
| **Residence region**  Westrobothnia  Ostrobothnia  South Ostrobothnia  Åland | 1  **0.68 (0.56-0.84)**  **0.79 (0.64-0.98)**  1.08 (0.79-1.47) | **<0.001**  **0.030**  0.622 | 1  **0.76**  0.82  1.11 | **0.61-0.94**  0.66-1.02  0.80-1.52 | **0.010**  0.078  0.534 | 1  **0.76**  0.83  1.09 | **0.62-0.94**  0.67-1.04  0.79-1.51 | **0.012**  0.102  0.591 | 1  **0.77**  0.82  1.08 | **0.62-0.95**  0.66-1.03  0.78-1.49 | **0.017**  0.091  0.651 |
| **Religiousness**  No/Passive  Active | 1  **0.26 (0.17-0.40)** | **<0.001** |  |  |  | 1  **0.30** | **0.19-0.46** | **<0.001** | 1  **0.30** | **0.19-0.46** | **<0.001** |
| **Loss of loved ones**  No  Yes | 1  0.96 (0.75-1.22) | 0.724 |  |  |  | 1  0.99 | 0.77-1.26 | 0.928 | 1  0.98 | 0.77-1.25 | 0.874 |
| **Loneliness**  Not lonely  Lonely | 1  1.09 (0.82-1.45) | 0.554 |  |  |  | 1  1.02 | 0.74-1.42 | 0.887 | 1  1.01 | 0.73-1.40 | 0.948 |
| **Inner strength scale** | 1.00 (0.99-1.00) | 0.239 |  |  |  | 0.99 | 0.99-1.00 | 0.083 | 0.99 | 0.99-1.00 | 0.073 |
| **Sleep quality**  Bad quality  Good quality | 1  **0.72 (0.61-0.86)** | **<0.001** |  |  |  | 1  **0.73** | **0.61-0.88** | **0.001** | 1  **0.75** | **0.62-0.90** | **0.002** |
| **Depression (GDS ≥2)**  No  Yes | 1  1.29 (0.98-1.69) | 0.065 |  |  |  | 1  1.08 | 0.79-1.47 | 0.639 | 1  1.07 | 0.78-1.47 | 0.685 |
| **CVD**  No  Yes | 1  **1.26 (1.05-1.51)** | **0.013** |  |  |  |  |  |  | 1  **1.32** | **1.08-1.61** | **0.007** |
| **Polypharmacy**  No  Yes | 1  0.85 (0.70-1.02) | 0.085 |  |  |  |  |  |  | 1  0.85 | 0.68-1.06 | 0.155 |
| **ADL independence**  No  Yes | 1  **1.50 (1.27-1.77)** | **<0.001** |  |  |  |  |  |  | 1  **1.31** | **1.09-1.57** | **0.004** |
| **Frailty**  Not frail  Frail | 1  1.03 (0.86-1.23) | 0.743 |  |  |  |  |  |  | 1  1.23 | 0.98-1.55 | 0.073 |
| **Physical activity**  <150 min/week  ≥150 min/week | 1  0.94 (0.79-1.12) | 0.491 |  |  |  |  |  |  | 1  0.92 | 0.75-1.12 | 0.377 |
| **BMI**  <23 kg/m^2^  23-29.9 kg/m^2^  ≥30 kg/m^2^ | 1  **1.58 (1.22-2.06)**  **2.36 (1.76-3.17)** | **0.001**  **<0.001** |  |  |  |  |  |  | 1  **1.54**  **2.08** | **1.17-2.02**  **1.52-2.84** | **0.002**  **<0.001** |
| **Subjective health**  Fair/bad  Good  Very good/excellent | 1  1.08 (0.89-1.32)  1.01 (0.83-1.22) | 0.442  0.953 |  |  |  |  |  |  | 1  1.10  1.08 | 0.87-1.38  0.84-1.40 | 0.445  0.545 |

# **Supplementary Table S6.** ORs, 95% confidence intervals (CIs), and p-values from ordinary logistic regression models for heavy episodic drinking among women.

| **Covariates** | **Model 1 (Bivariate)** | | **Model 2 (Sociodemographic)** | | | **Model 3 sociodemographic +**  **other psychosocial functioning** | | | **Model 4 sociodemographic +**  **other psychosocial variables +**  **Physical health/physical functioning** | | |
| --- | --- | --- | --- | --- | --- | --- | --- | --- | --- | --- | --- |
|  | **OR (95%CI)** | **p value** | **OR** | **95%CI** | **p value** | **OR** | **95%CI** | **p value** | **OR** | **95%CI** | **p value** |
| **Age group**  65  70  75  80  85  90 | 1  0.91 (0.64-1.30)  **0.57 (0.37-0.87)**  **0.46 (0.25-0.82)**  **0.11 (0.03-0.45)**  **0.11 (0.02-0.81)** | 0.611  **0.009**  **0.009**  **0.002**  **0.031** | 1  0.97  **0.61**  **0.49**  **0.13**  **0.14** | 0.67-1.39  **0.39-0.94**  **0.27-0.91**  **0.03-0.52**  **0.02-1.00** | 0.854  **0.025**  **0.023**  **0.004**  **0.050** | 1  0.97  **0.61**  **0.47**  **0.12**  **0.13** | 0.67-1.40  **0.40-0.95**  **0.26-0.88**  **0.03-0.49**  **0.02-0.93** | 0.866  **0.028**  **0.017**  **0.003**  **0.042** | 1  0.98  **0.61**  **0.47**  **0.11**  **0.12** | 0.68-1.41  **0.39-0.94**  **0.25-0.87**  **0.03-0.48**  **0.02-0.88** | 0.894  **0.026**  **0.020**  **0.003**  **0.037** |
| **Marital status**  Single/divorced/widow  Married/partner | 1  1.37 (0.99-1.90) | 0.057 | 1  1.09 | 0.78-1.53 | 0.607 | 1  1.24 | 0.88-1.76 | 0.225 | 1  1.24 | 0.87-1.76 | 0.234 |
| **Education level**  Primary/ lower 2ndary  Upper secondary  Higher education | 1  1.06 (0.73-1.53) **1.77 (1.20-2.61)** | 0.765  **0.004** | 1  0.83  1.14 | 0.57-1.22  0.74-1.75 | 0.347  0.559 | 1  0.88  1.30 | 0.60-1.29  0.84-2.01 | 0.513  0.241 | 1  0.88  1.26 | 0.60-1.29  0.81-1.96 | 0.503  0.313 |
| **Income**  ≤1000 €  1001-2000 €  ˃2000 € | 1  1.30 (0.85-1.98)  **2.12 (1.19-3.76)** | 0.225  **0.010** | 1  1.14  1.37 | 0.74-1.77  0.73-2.57 | 0.553  0.328 | 1  1.20  1.55 | 0.77-1.86  0.82-2.93 | 0.415  0.177 | 1  1.12  1.54 | 0.77-1.87  0.81-2.93 | 0.422  0.191 |
| **Residence region**  Westrobothnia  Ostrobothnia  South Ostrobothnia  Åland | 1  **0.68 (0.47-1.00)**  **0.57 (0.37-0.88)**  **1.73 (1.09-2.76)** | **0.048**  **0.012**  **0.021** | 1  0.78  0.67  **1.80** | 0.53-1.16  0.43-1.04  **1.12-2.92** | 0.223  0.077  **0.016** | 1  0.81  0.68  **1.81** | 0.55-1.21  0.44-1.07  **1.12-2.94** | 0.306  0.099  **0.016** | 1  0.85  0.72  **1.86** | 0.56-1.26  0.45-1.13  **1.14-3.03** | 0.412  0.150  **0.013** |
| **Religiousness**  No/Passive  Active | 1  **0.40 (0.22-0.74)** | **0.003** |  |  |  | 1  **0.48** | **0.26-0.90** | **0.021** | 1  **0.49** | **0.27-0.91** | **0.024** |
| **Loss of loved ones**  No  Yes | 1  1.36 (0.96-1.91) | 0.083 |  |  |  | 1  **1.51** | **1.06-2.14** | **0.022** | 1  **1.51** | **1.06-2.15** | **0.022** |
| **Loneliness**  Not lonely  Lonely | 1  1.18 (0.77-1.82) | 0.446 |  |  |  | 1  1.13 | 0.69-1.86 | 0.635 | 1  1.13 | 0.68-1.86 | 0.644 |
| **Inner strength scale** | **0.99 (0.98-1.00)** | **0.022** |  |  |  | **0.98** | **0.97-0.99** | **0.003** | **0.98** | **0.97-1.00** | **0.004** |
| **Sleep quality**  Bad quality  Good quality | 1  0.81 (0.59-1.10) | 0.168 |  |  |  | 1  0.87 | 0.63-1.19 | 0.379 | 1  0.84 | 0.61-1.17 | 0.298 |
| **Depression (GDS ≥2)**  No  Yes | 1  **1.66 (1.09-2.51)** | **0.018** |  |  |  | 1  1.37 | 0.84-2.23 | 0.206 | 1  1.44 | 0.87-2.37 | 0.155 |
| **CVD**  No  Yes | 1  0.88 (0.65-1.19) | 0.413 |  |  |  |  |  |  | 1  1.16 | 0.83-1.62 | 0.380 |
| **Polypharmacy**  No  Yes | 1  **0.52 (0.34-0.80)** | **0.003** |  |  |  |  |  |  | 1  **0.58** | **0.36-0.95** | **0.029** |
| **ADL independence**  No  Yes | 1  1.28 (0.92-1.78) | 0.146 |  |  |  |  |  |  | 1  0.97 | 0.67-1.41 | 0.859 |
| **Frailty**  Not frail  Frail | 1  0.96 (0.71-1.30) | 0.799 |  |  |  |  |  |  | 1  1.19 | 0.81-1.76 | 0.383 |
| **Physical activity**  <150 min/week  ≥150 min/week | 1  0.88 (0.65-1.19) | 0.413 |  |  |  |  |  |  | 1  **0.66** | **0.47-0.94** | **0.021** |
| **BMI**  <23 kg/m^2^  23-29.9 kg/m^2^  ≥30 kg/m^2^ | 1  1.00 (0.69-1.46)  0.80 (0.49-1.30) | 0.999  0.371 |  |  |  |  |  |  | 1  0.99  0.79 | 0.68-1.46  0.47-1.33 | 0.975  0.370 |
| **Subjective health**  Fair/bad  Good  Very good/excellent | 1  1.28 (0.87-1.89)  1.35 (0.93-1.95) | 0.211  0.114 |  |  |  |  |  |  | 1  1.24  1.43 | 0.80-1.94  0.88-2.35 | 0.342  0.153 |
